# NIR-activated electrospun nanodetonator dressing enhances infected diabetic wound healing with combined photothermal and nitric oxide-based gas therapy

**DOI:** 10.1186/s12951-024-02474-9

**Published:** 2024-05-08

**Authors:** Jiajun Xie, Guihua Liu, Rong Chen, Ding Wang, Huaming Mai, Qiang Zhong, Yanhong Ning, Jinlang Fu, Zinan Tang, Yixin Xu, Hao Li, Mingyuan Lei, Hao Cheng, Yuliang Huang, Yang Zhang

**Affiliations:** 1 Division of Orthopaedic Surgery, Department of Orthopaedics, Nanfang Hospital, Southern Medical University, Guangzhou, 510515 Guangdong People’s Republic of China; 2 Institute of Orthopaedics, Huizhou Central People’s Hospital, Huizhou, 516008 Guangdong People’s Republic of China

**Keywords:** S-Nitrosoglutathione, Nanofiber membrane, Photothermal antibacterial therapy, Nitric oxide shattering, Multifunctional electrospun dressing, Diabetic wound healing

## Abstract

Diabetic wounds pose a challenge to healing due to increased bacterial susceptibility and poor vascularization. Effective healing requires simultaneous bacterial and biofilm elimination and angiogenesis stimulation. In this study, we incorporated polyaniline (PANI) and S-Nitrosoglutathione (GSNO) into a polyvinyl alcohol, chitosan, and hydroxypropyltrimethyl ammonium chloride chitosan (PVA/CS/HTCC) matrix, creating a versatile wound dressing membrane through electrospinning. The dressing combines the advantages of photothermal antibacterial therapy and nitric oxide gas therapy, exhibiting enduring and effective bactericidal activity and biofilm disruption against methicillin-sensitive *Staphylococcus aureus*, methicillin-resistant *Staphylococcus aureus*, and *Escherichia coli*. Furthermore, the membrane’s PTT effect and NO release exhibit significant synergistic activation, enabling a nanodetonator-like burst release of NO through NIR irradiation to disintegrate biofilms. Importantly, the nanofiber sustained a uniform release of nitric oxide, thereby catalyzing angiogenesis and advancing cellular migration. Ultimately, the employment of this membrane dressing culminated in the efficacious amelioration of diabetic-infected wounds in Sprague–Dawley rats, achieving wound closure within a concise duration of 14 days. Upon applying NIR irradiation to the PVA-CS-HTCC-PANI-GSNO nanofiber membrane, it swiftly eradicates bacteria and biofilm within 5 min, enhancing its inherent antibacterial and anti-biofilm properties through the powerful synergistic action of PTT and NO therapy. It also promotes angiogenesis, exhibits excellent biocompatibility, and is easy to use, highlighting its potential in treating diabetic wounds.

**Graphical Abstract:**

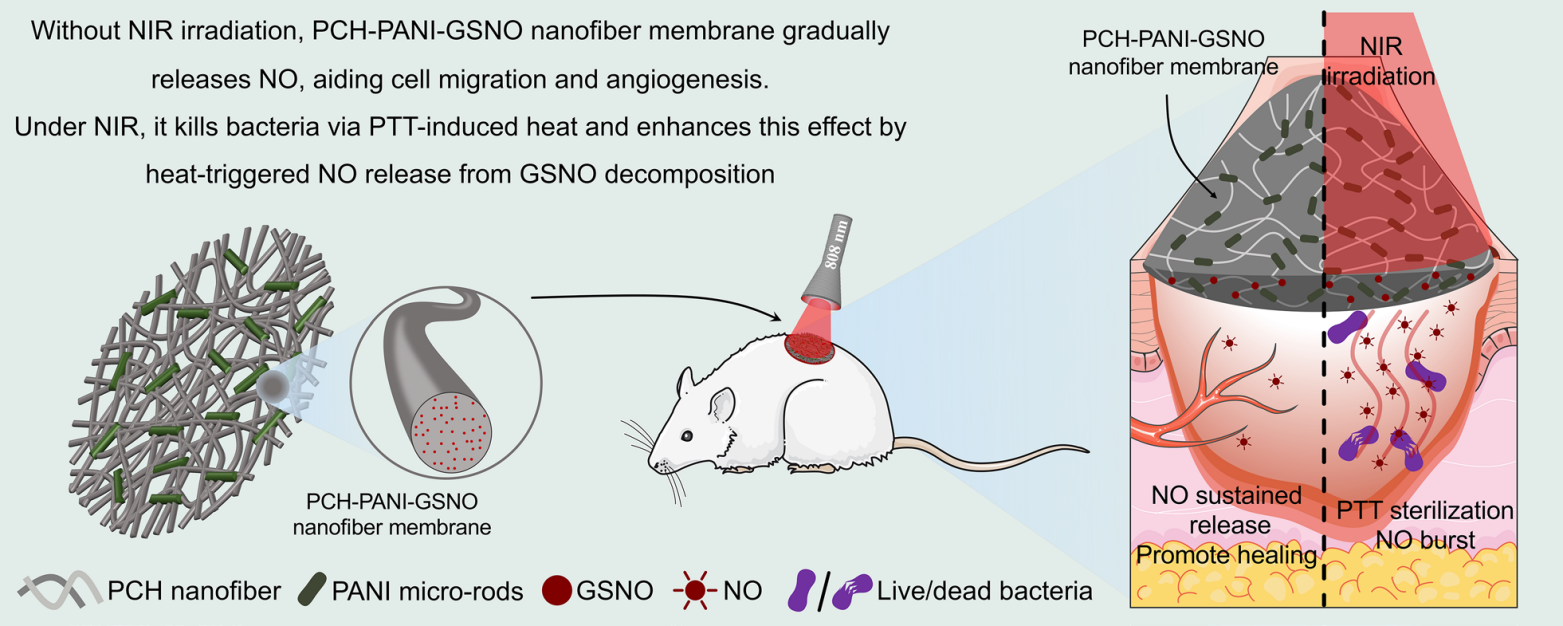

**Supplementary Information:**

The online version contains supplementary material available at 10.1186/s12951-024-02474-9.

## Introduction

Globally, diabetes is estimated to afflict 382 million adults, comprising 8.3% of the population, and markedly increases the prevalence of chronic wounds, thereby escalating morbidity, mortality, and healthcare costs [1]. A substantial proportion of individuals with diabetes, approximately 19–34%, encounter a heightened risk of developing chronic wounds, such as foot ulcers, leading to persistent discomfort and potential amputation in severe instances [2]. Wound healing is a dynamic and highly regulated process that regenerates injured skin through four overlapping stages: hemostasis, inflammation, proliferation, and remodeling [3]. Diabetic wounds (DW) struggle to smoothly progress through these stages due to the hyperglycemic environment’s damage to endothelial function, autonomic neuropathy’s destruction of skin’s self-regulation, anemia and hypoxia caused by microangiopathy, stubborn infections from various bacteria due to barrier disruption, defects in the local immune system, and the impact of psychosocial factors [4]. The predominant strategy for managing chronic diabetic wounds involves systematic debridement and routine changes of dressings [5]. However, traditional wound dressings often fall short in addressing the susceptibility to polymicrobial infections, bacterial biofilm formation, and impaired local blood circulation, consequently resulting in delayed wound healing [6]. Thus, the development of wound dressings that offer robust antimicrobial defense and enhance local blood circulation is crucial for efficacious wound healing. Various functional materials (Hydrogels, foams, and films, etc.) for wound dressings have been engineered to thwart infection and facilitate wound closure [7]. Nevertheless, the antibacterial efficacy of contemporary wound dressings is constrained by their narrow-spectrum activity and transient duration. Moreover, these dressings only offer short-term enhancements in blood perfusion.

Nanofiber membranes employed in wound dressings have attracted interest due to their superior hydrophilicity, permeability to gases, and elevated porosity [8]. These membranes, characterized by an exceptionally large specific surface area, enable the meticulous modulation of drug release kinetics while maintaining the integrity of drug encapsulation [9]. Techniques employed in the fabrication of nanofiber membranes encompass electrospinning, a process leveraging electrostatic forces; phase separation, utilizing differential solubility; template synthesis, based on structured molds; and self-assembly, driven by molecular interactions [10]. Notably, the versatility of electrospinning stands out, especially in terms of material selection and preparative techniques. This method facilitates the precise integration and delivery of a wide array of polymers and therapeutic agents [11]. As a result, electrospun membranes are adept at seamlessly incorporating bioactive agents that possess essential antibacterial properties and enhance blood perfusion, thereby significantly speeding up the wound healing process [12].

As a natural cationic polysaccharide derived from the exoskeletons of crustaceans and insects, chitosan (CS) inherently possesses excellent biocompatibility and biodegradability. Consequently, CS is also widely applied in the construction of drug delivery hydrogels, cell scaffolds, and electrospun biomaterials [13]. The hydrophilic properties of polyvinyl alcohol (PVA) have led to its integration into CS matrices as a secondary polymer to enhance the electrospinning process. In the present investigation, hydroxypropyltrimethyl ammonium chloride chitosan (HTCC) was additionally incorporated into the composite matrix to imbue the membrane with intrinsic and sustained antibacterial capabilities.

Nonetheless, although HTCC predominantly protects the wound via a contact-mediated bactericidal mechanism, there exists a potential inadequacy in comprehensively eliminating bacteria in the wound vicinity. Open dermal wounds are often susceptible to colonization by a multitude of bacteria, including *Escherichia coli* and *Staphylococcus aureus* [14]. An inability to achieve immediate sterilization may facilitate the establishment of biofilms and the proliferation of intracellular bacterial colonies, thereby complicating the process of bacterial eradication [15].

As a result, the principal antibacterial mechanism of dressings designed for DW necessitates rapid, wide-spectrum sterilization coupled with the ability to disrupt biofilm formations [16]. Approaches such as the implementation of photothermal antibacterial therapy (PTT) have been proposed to counteract wound-associated pathogens. PTT leverages photothermal agents to transduce light energy into localized thermal energy, facilitating the disassembly of bacterial and biofilm architectures, thereby either inducing direct bacterial cytotoxicity or enhancing the performance of established antibacterial systems [17]. Moreover, the antibacterial modality of PTT presents benefits such as a diminished likelihood of engendering drug resistance and prompt, efficacious bacterial eradication [18].

Polymeric materials for PTT have now received widespread attention [19]. Among them, PANI has garnered interest due to its excellent photothermal properties, electrical conductivity, mechanical flexibility, and cost-effectiveness [20]. Moreover, PANI demonstrates negligible cytotoxicity and has been successfully utilized as an electroactive substrate in cell proliferation studies [21]. Additionally, in drug delivery systems, the PTT effect can be utilized to increase the rate of drug delivery [22, 23].

To facilitate the efficacious dispersion of established mature biofilms, S-Nitrosoglutathione (GSNO), a stable nitric oxide (NO) donor, was integrated into the membrane matrix. GSNO is capable of consistently releasing NO under physiological conditions and exhibits accelerated NO liberation in response to thermal stimuli [24]. In synergy with photothermal therapy (PTT) materials under light irradiation, such as near-infrared (NIR), photothermal agents such as PANI can convert light energy into thermal energy, thereby causing GSNO to promptly react, releasing substantial quantities of NO, and thus effectuating a rapid eradication of bacteria and biofilms [25].

Crucial to infection control is the enhancement of local blood circulation and the stimulation of cellular migration, both imperative for diabetic wound healing [26]. Dissolved NO, known for its capacity to induce vasodilation and angiogenesis, promotes the expansion and movement of fibroblasts, thus accelerating the process of wound healing [27]. Additionally, encapsulating GSNO within polymeric matrices can modulate its decomposition rate, ensuring a sustained release of NO, a feature pivotal during the healing trajectory [28].

In this study, a composite PVA-CS-HTCC-PANI-GSNO electrospun nanofiber membrane was engineered, comprising a matrix of PVA, CS, and HTCC, supplemented with PANI and GSNO for photothermal and NO release functionalities, respectively. The devised dressing amalgamates attributes essential for effective wound management, such as swift and broad-spectrum antibacterial activity, controlled multi-modal antibacterial properties, and simultaneous promotion of local angiogenesis and fibroblast migration. Thorough evaluations of the membrane’s photothermal properties, NO release kinetics, antibacterial effectiveness, and biocompatibility were performed using both in vitro and in vivo approaches. These assessments highlight the capability of our multifunctional dressing to address the clinical and financial challenges related to the treatment of infectious DW.

## Experimental section

### Materials

Poly(vinyl alcohol) (PVA 1788, MW ~ 67,000, 87–90% hydrolyzed, CAS: 9002–89-5), chitosan (CS, average MW = 50,000, ≥ 95% deacetylated, CAS: 9012-76-4), hydroxypropyltrimethyl ammonium chloride chitosan (HTCC, MW = 10,000, degree of substitution = 98%, MACKLIN Shanghai, China), sodium dodecylbenzenesulfonate (MW 348.48, purity: 95%, formula: C_18_H_29_NaO_3_S, CAS: 25155-30-0), aniline (MW 93.13, purity ≥ 99.5%, formula: C_6_H_7_N, CAS:), hydrochloric acid (HCl, 37% w/w, CAS: 62–53-3), ammonium persulfate (MW 228.2, AR, 98.5%, formula: H_8_N_2_O_8_S_2_, CAS: 7727-54-0), S-Nitrosoglutathione (GSNO, MW = 336.32, purity: 95%, formula: C_10_H_16_N_4_O_7_S, CAS: 57564-91-7), glutaraldehyde (GA, MW 100.12, 25% in H_2_O, formula: C_5_H_8_O_2_, CAS: 111-30-8), sodium nitrite (MW 69, 99% metals basis, formula: NaNO_2_, CAS: 7632-00-0), 4-Amino-5-Methylamino-2′,7′-Difluorofluorescein Diacetate (DAF-FM DA, MW 496.42, purity: 98%, formula: C_25_H_18_F_2_N_2_O_7_, CAS: 254109-22-3), crystal violet (MW 407.98, formula: C_25_H_30_N_3_Cl, CAS: 548-62-9), Streptozotocin (STZ, MW 265.22, purity: 98%, formula: C_8_H_15_N_3_O_7_, CAS: 18883-66-4), acetic acid (MW = 60.05, purity: 99.7%, formula: C_2_H_4_O_2_, CAS: 64-19-7), anhydrous ethanol (MW = 46.07, purity: 99.5%, formula: C_2_H_6_O, CAS: 64--17-5). iFluor 488 phalloidin, 4′,6-diamidino-2-phenylindole (DAPI) and VEGF (vascular endothelial growth factor) were obtained from YEASEN (Shanghai, China). Phosphate buffered saline (PBS), DMEM, cell counting kit-8 (CCK-8), Luria–Bertani (LB) broth, fetal bovine serum, paraformaldehyde, Matrigel, agar plates were obtained from MACKLIN (Shanghai, China). Cell and bacterial live/dead staining kit was obtained from Bestbio (Shanghai, China). Griess reagent (G4410-10G) was obtained from Sigma-Aldrich (USA). L929 rat cells and human umbilical vein endothelial cells (HUVECs) were obtained from Cyagen (Jiangsu, China). ATCC (Manassas, VA, USA) supplied *E. coli* (*Escherichia coli*, ATCC 252922), MSSA (methicillin-sensitive *Staphylococcus aureus*, ATCC 25923), and MRSA (methicillin-resistant *Staphylococcus aureus*, ATCC 43300). The experimental apparatus involved in this study are as follows: lyophilizer (CoolSafe110-4, LABOGENE, Denmark), high-voltage power supply unit (SIBEINING, China), drum collector (SIBEINING, China), syringe pump (SIBEINING, China), vacuum chamber (TENGYUAN, Zhejiang, China), scanning electron microscope (SEM) (Sigma 300, ZEISS, Germany), Fourier Transform Infrared Spectrometer (TENSOR 27, BRUKER, Germany), Thermogravimetric Analyzer (TGA/DSC3 +, Mettler Toledo, USA), inclined contact-angle measurement device (DSA-XROLL, BETOP SCIENTIFIC, China), 808 nm near infrared (NIR) laser (LR-ISP-808, LASER TECHNOLOGY, China), infrared thermal imaging camera (UTi 320E, UNI-T, China), microplate reader (SpectraMax i3x, MOLECULAR DEVICES, Germany), inverted fluorescence/phase-contrast microscope (ECLIPSE Ti2-E, Nikon, Japan), and Bayer Contour Next EZ instrument (Leverkusen, Germany).

### Methods

#### Synthesis of PANI rods

A 200 mL round-bottom flask was charged with 120 mL of deionized water, 0.16 g of sodium dodecylbenzenesulfonate, and 0.8 mL of aniline. The constituents were homogenized via magnetic stirring within an ice bath for a duration of 30 min. Subsequent to this, 5.44 mL of hydrochloric acid (HCl) was introduced to the flask. Concurrently, a secondary solution was prepared by dissolving 1 g of ammonium persulfate and 1.84 mL of HCl in 40 mL of deionized water. This secondary solution was incrementally introduced to the primary reaction milieu over a span of 30 min. The amalgamated solution was then subjected to continuous stirring in the ice bath for an additional 8 h. The resultant green PANI rod powder was isolated by subjecting the final product to multiple washings with deionized water, culminating in lyophilization. The synthesis steps for PANI rods are illustrated in Additional file 1: Fig. S1.

#### Preparation of the prepolymer solution

Initially, a solution was prepared by dissolving 800 mg of PVA (80% w/w), 150 mg of CS (15% w/w), and 50 mg of HTCC (5% w/w) in 8 mL of a 1% (v/v) acetic acid solution. We used covered sample vials for heating and stirring the polymer solution at a rotational speed of 500 rpm and a controlled temperature of 45 °C for 12 h, which prevents the evaporation of the solvent. Subsequently, 5 mg of PANI was evenly distributed within 1 mL of trifluoroethanol using a water bath ultrasonicator (Scientz, China, with ultrasonic power set at 200W and frequency at 40 kHz) for a continuous treatment of 180 s. In a subsequent step, 15 mg of GSNO was solubilized in 1 mL of bi-distilled H_2_O. The final synthesis stage entailed the amalgamation of the three aforementioned solutions at a condition of 25 °C, followed by stirring at a rotational speed of 500 rpm for a period of 4 h.

#### Electrospinning and crosslinking of nanofibrous membrane

**Fig. 1:**
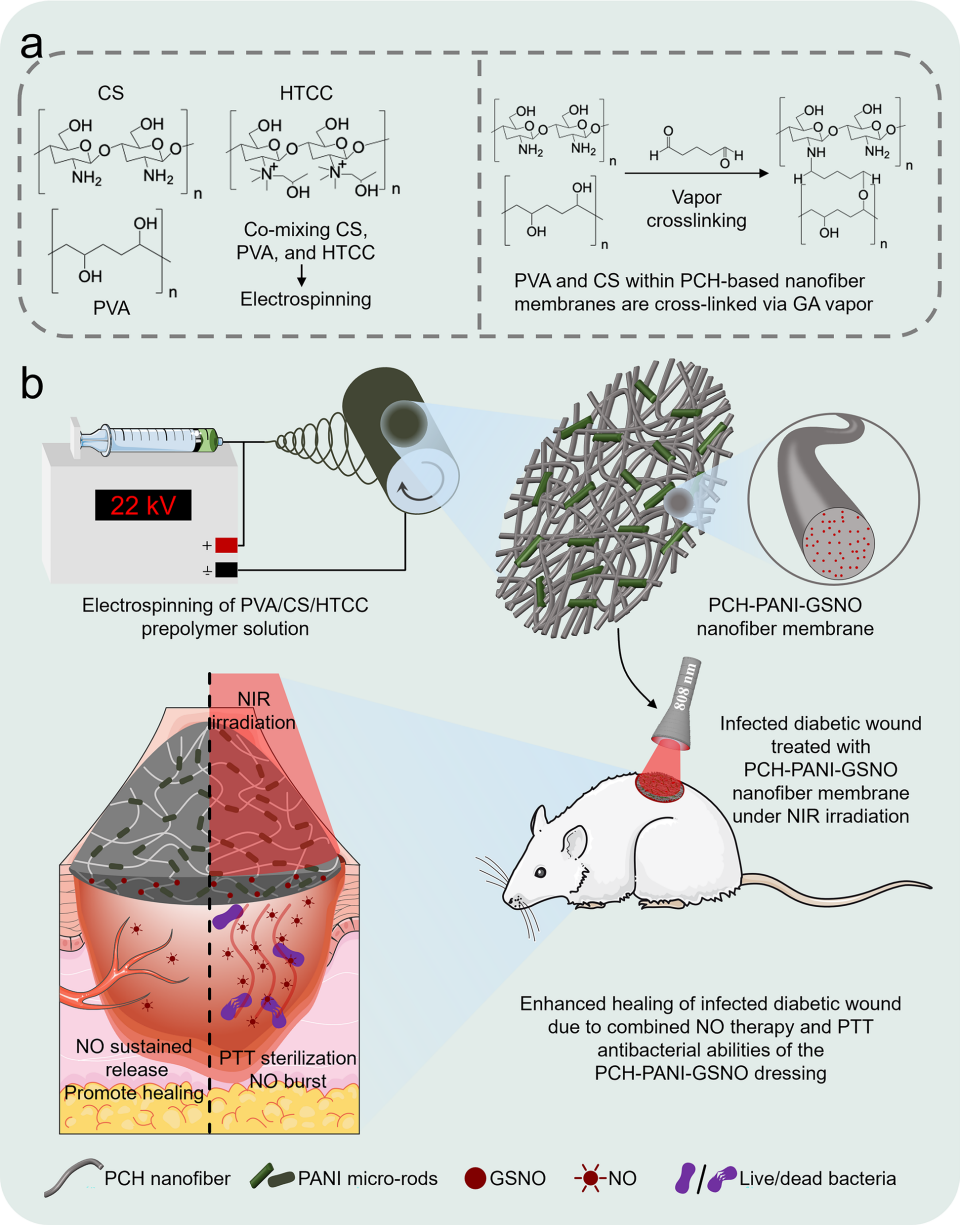
Schematic of the synthesis and functional expression process of PCH-based nanofiber membranes. **a** Diagram showcasing the cross-linking between PVA and CS within PCH electrospun nanofibers using GA vapor. **b** Depiction of how the PCH-PANI-GSNO nanofiber membrane aids in healing infectious DW

The composite prepolymer formulations, designated as PVA-CS-HTCC (PCH), PVA-CS-HTCC-GSNO (PCH-GSNO), PVA-CS-HTCC-PANI (PCH-PANI), and PVA-CS-HTCC-PANI-GSNO (PCH-PANI-GSNO), each with a volume of 10 mL, were loaded into a 10 mL syringe fitted with a 22-gauge stainless-steel needle. The syringe was connected to the electrode of a high-voltage power supply unit (SIBEINING, China). Concurrently, the grounding wire was affixed to a drum collector rotating at a speed of 500 rpm. The electrospinning solution was extruded at a controlled flow rate of 5 μL/min via a precision syringe pump (SIBEINING, China). A gap of 16.5 cm was maintained between the needle tip and the collector, with the power supply unit set at a voltage of 22 kV. Subsequently, the resulting nanofibrous membranes were subjected to a vapor-phase crosslinking treatment using a mixture of glutaraldehyde (GA) and HCl for a duration of 1 h at a pressure of 6 × 10^–2^ MPa within a vacuum chamber. The vapor-phase crosslinking agent was generated from 1 mL of 50 wt% GA and 20 µL of 37 wt% HCl in water, with HCl catalyzing the formation of acetal bridges between the hydroxyl groups of PVA and the aldehyde groups of GA. Post crosslinking, the membranes were allowed to air-dry in a fume hood for 24 h to ensure the complete evaporation of residual GA and HCl. Circular specimens, with a diameter of 1 cm (10 mg in weight), were cut from the collected nanofiber membranes using a circular punch. The thickness of the nanofiber membranes was measured using a micrometer (GREENER, China) to ensure uniform thickness of the samples. Subsequently, these samples were used for further experimental analysis. The preparation of the prepolymer solution and the fabrication of electrospun nanofiber membranes are detailed in Additional file 1: Fig. S2. Figure 1 provides a schematic representation of the operational process of nanofiber membranes.

#### SEM analysis of the nanofibrous membrane and PANI rods

To study the morphology of the nanoelectrospun fibers, a scanning electron microscope (SEM) (Sigma 300, ZEISS, Germany) was used to obtain microstructural images of the nanofiber membranes. The procedure is outlined as follows: PCH-based nanofiber membranes are affixed to the surface of an aluminum sample stage using conductive adhesive, then transferred to a sputtering device (ETD-2000C, BOYUANWEINA, China). After evacuating the chamber, gold sputtering is performed at a current of 10 mA for 120 s. Subsequently, the sample is moved to the SEM chamber, where, after vacuum application, imaging is conducted using the inlens detector at a working voltage of 15 kV, with an aperture of 30 μm and a working distance of 8.5 mm, to observe the sample surface's morphology at various magnifications. For mapping nitrogen elements, the equipped Bruker Quantax 6/30 is activated to collect elemental signals under a 60 μm aperture and a high beam energy of 20 kV, thereby generating corresponding elemental distribution maps. For the SEM observation of PANI rods, the method involves dispersing the PANI rods on aluminum foil using trifluoroethanol and then observing them in the same manner after drying the trifluoroethanol. The diameter distribution of the nanofibers, the distribution of the major and minor axes of the PANI rods, and the porosity of the nanofiber membranes were analyzed using ImageJ software (v1.53).

#### FT-IR analysis of the nanofibrous membrane

A Fourier Transform Infrared Spectrometer (TENSOR 27, BRUKER, Germany) was utilized to elucidate both the chemical structure and the types of surface functional groups of PCH-based nanofiber membranes. The membrane samples were directly assessed using an ATR (Attenuated Total Reflectance) attachment. This approach involved invoking the reflection mode to acquire the ATR-FTIR (Fourier-transform infrared spectroscopy) spectra with full reflection of the samples. The spectral range spanned from 4000 to 600 cm^−1^, with a resolution of 2 cm^−1^ for each wavenumber step.

#### Thermogravimetric analysis and contact angle analysis

After synthesizing the PCH-based nanofiber membranes, the moisture was removed using a lyophilizer (CoolSafe110-4, LABOGENE, Denmark). Subsequently, thermogravimetric examination of the electrospun nanofibers was conducted utilizing a Thermogravimetric Analyzer (TGA/DSC3 +, Mettler Toledo, USA). The test temperature range was set from 30 to 800 °C, with a heating rate of 10 °C/min, under an atmosphere of nitrogen gas flowing at a rate of 50 mL/min.

Optical water contact angles were utilized to evaluate the wettability of the nanofiber membranes. This was conducted using an inclined contact-angle measurement device (DSA-XROLL, BETOP SCIENTIFIC, China). Specifically, 3 μL of deionized water was gently placed onto the membrane. Subsequent to this, images were captured using a high-resolution camera, depicting the static state of the liquid deposition. Replicate measurements (a minimum of three per sample) were performed, and the average figure was computed to obtain the contact angle.

#### Water absorption and degradation rate assessment of nanofiber membranes

The membrane specimens were divided into PCH, PCH-GSNO, PCH-PANI, PCH-PANI-GSNO, and PCH-PANI + NIR groups, and were then initially desiccated in a vacuum oven set for a 12-h duration, after which their weights were determined using a micro-scale. The determined dry weight was 10 mg, indicated as M_0_. The specimens were then immersed in 10 mL of PBS for durations of 3, 6, 12, 24, 48, and 72 h. During this period, samples from the PCH-PANI-GSNO + NIR group were subjected to 5 min of NIR on/off cyclic irradiation at a power density of 0.5 W/cm^2^ at the 0th and 48th hours (marking the beginning of the 1st and 3rd days). These samples were subsequently reweighed, and the weights were recorded as M_1_. To calculate the water absorption ratio of the sample, the following formula was employed [29]:

1 $${water\,\,absorption\,\, ratio = [M_1 - M_0] /M0\# }$$

For assessing the nanofibers’ degradation rate in vitro, the specimens were submerged in 10 mL PBS and maintained at 37 °C over 14 days. The samples were divided into PCH, PCH-GSNO, PCH-PANI, PCH-PANI-GSNO, and PCH-PANI-GSNO + NIR groups. The samples were extracted from the PBS solution at specific intervals (0, 1, 3, 5, 7, and 14 days). During this period, samples from the PCH-PANI + NIR group were subjected to 5 min of NIR on/off cyclic irradiation at a power density of 0.5 W/cm^2^ at the onset of 1, 3, 5, 7, 9, 11, and 13 days (which corresponds to 0, 48, 96, 144, 192, 240, 288 h), followed by removal of surplus moisture using a lyophilizer (CoolSafe110-4, LABOGENE, Denmark). Each sample’s weight was noted as (W_x_) and contrasted with its initial weight (10 mg, W_o_). The subsequent formula was used to determine the degradation rate [30]:

2 $$degradation\, rate = \left[ {\left( {{W_o}{W_x}} \right)/{W_o}} \right] \times 100\%$$

Water absorption and degradation rate assessment for all samples were repeated three times.

#### Photothermal properties of nanofiber membrane

The PCH-PANI-GSNO nanofibrous membrane was exposed to an 808 nm laser (LR-ISP-808, LASER TECHNOLOGY, China) irradiation at a power density of 0.5 W/cm^2^ for a duration of 120 s. To determine the NIR laser power density on the nanofiber membranes, we set the laser output power at 0.5 W and adjusted the fiber optic collimator of the 808 nm laser (ISP-808, LASER TECHNOLOGY, China) to produce a light spot diameter of 1.13 cm, corresponding to a spot area of 1 cm^2^. Thus, the power density was maintained at the specified 0.5 W/cm^2^. The temporal evolution of temperature was monitored using an infrared thermal imaging camera (UTi 320E, UNI-T, China). To evaluate the photothermal properties, PBS, PCH, PCH-PANI, and PCH-PANI-GSNO were examined under identical experimental conditions. During the on/off cycles assessment, the specimen was subjected to NIR irradiation for 25 s, with each successive cycle commencing once the temperature returned to ambient levels (25 °C), approximately within 30 s. In all subsequent experiments, we adopted this uniform NIR on/off cyclic irradiation at a power density of 0.5 W/cm^2^ for NIR exposure of cells, bacteria, and animals in groups requiring NIR exposure, with each exposure lasting for 5 min.

#### NO release and NIR-responsive NO burst from PCH-PANI-GSNO

The sustained-release properties of NO from PCH-PANI-GSNO were assessed by immersing a 10 mg sample of the nanofibrous membrane in 2 mL of PBS at 37 °C. The NO concentration in the PBS was quantified bi-daily up to day 14 using Griess reagent (G4410-10G; Sigma-Aldrich). A succinct method involves taking 50μL of PBS that had been immersed with the nanofiber membrane at various time points and thoroughly mixing it with an equal volume of Griess reagent in a 96-well plate. After incubating the 96-well plate at 37 °C in the dark for 10 min, the absorbance at 540 nm was measured using a microplate reader (SpectraMax i3x, MOLECULAR DEVICES, Germany). The amount of NO released from the nanofiber membrane was determined by comparing the obtained absorbance with that of a standard sodium nitrite solution measured using the same method. Additionally, to examine the NIR-induced NO burst release, an equivalent mass of the sample was submerged in 2 mL of PBS and subjected to the aforementioned NIR on/off cyclic irradiation at a power density of 0.5 W/cm^2^, with exposures of 5 min at 55-min intervals. The NO concentration in the PBS was consistently quantified using the previously described method. The photothermal properties of each sample were measured three times.

#### Cell culture in vitro

L929 cells, sourced from populations prior to the fifth passage, were utilized to evaluate the biocompatibility of nanofibrous membranes. These cells were propagated in DMEM supplemented with 10% fetal bovine serum, under conditions of 5% CO_2_ at 37 °C, with media renewal occurring bi-daily. The NIR exposure groups (control + NIR, PCH + NIR, PCH-GSNO + NIR, PCH-PANI + NIR, PCH-PANI-GSNO + NIR) were subjected to NIR irradiation at a wavelength of 808 nm and a power density of 0.5 W/cm^2^, in contrast to the non-NIR exposed groups (control, PCH, PCH-GSNO, PCH-PANI, PCH-PANI-GSNO). Each irradiation session lasted for 25 s, followed by a cooling period until the temperature returned to 37 °C before the next cycle, with the total duration being 5 min. To ensure consistency in NIR exposure during cell experiments, the on/off cyclic NIR exposure for each group was conducted after cell adhesion, at the time of nanofiber membranes placement. During a test spanning 3 days, NIR on/off cyclic irradiation was applied at the onset of 1, 3 days (which corresponds to 0, 48 h).

#### Cell viability and biocompatibility assay

In co-incubations incorporating membrane samples, L929 cell viability was assessed using the CCK-8 assay [31]. Under conditions of 37 °C and 5% CO_2_, 1 × 10^4^ cells were co-incubated with PCH-based nanofiber membranes for periods of both 6 and 12 h. Using a microplate reader (SpectraMax i3x, MOLECULAR DEVICES, Germany), the absorbance of the resulting supernatant was determined at a wavelength of 450 nm. For the CCK-8 assay, the cell viability was determined employing the following equation:

3 $${cell\,\, viability\,(\%) = [(OD_m - OD_b)/(OD_c - OD_b)] \times 100\% }$$

Within this framework, the OD_b_ is associated with DMEM with 10% fetal bovine serum. Conversely, the OD_c_ represents the spectral reading from the cellular solution. Simultaneously, the OD_m_ pertains to the solution derived from cell culture combined with nanofiber membranes.

A live/dead fluorescence staining kit (Bestbio, China) was employed in a live/dead fluorescence staining assay to assess the viability and cytotoxicity of L929 cells co-incubated with various groups. After co-incubation and NIR irradiation (to NIR exposure groups as described above) with the nanofiber membranes, cells underwent staining and a subsequent 20-min incubation in darkness. Thereafter, the samples were imaged utilizing a fluorescence microscope (ECLIPSE Ti2-E, Nikon, Japan). The fluorescent cells, green for live and red for dead, were quantified using ImageJ software (v1.53). The data thus obtained facilitated the determination of cell viability using the prescribed equation:

4 $${cell\, viability\,(\%) = [{N_{live}}/({N_{live}} + {N_{dead}})] \times 100\% }$$

In this context, N_live_ represents the count of live cells, while N _dead_ indicates the count of dead ones.

#### Cell morphology assay

To evaluate cellular morphology, L929 cells were stained with iFluor 488 phalloidin (YEASEN, China) and DAPI. Following the respective group treatments, L929 cells were deprived of their culture medium and subsequently underwent three gentle washes with PBS, followed by fixation with 4% paraformaldehyde. Subsequently, the cells were incubated with iFluor 488 phalloidin at 37 °C for 4 h and then co-incubated with DAPI for 5 min. After incubation, the cells were gently washed three times with PBS to remove excess stain. Upon completion of the staining procedure, cellular imaging was performed using an inverted fluorescence microscope (ECLIPSE Ti2-E, Nikon, Japan). The acquired images were analyzed using the ImageJ software (v1.53).

#### Intracellular NO detection

In an initial proof-of-concept investigation, L929 cells and nanofiber membranes were co-incubated in a 24-well plate for a time span of 6 h after undergoing respective group treatments. Subsequent to the co-incubation, the cell culture medium was meticulously removed. Each well was treated with 200 μL of DAF-FM DA (YEASEN, China) at a concentration of 5 mM. The assembly was then incubated at a controlled temperature of 37 °C for a duration of 20 min. Post incubation, the cells underwent three consecutive washes with PBS, and were subsequently examined under a fluorescence microscope (ECLIPSE Ti2-E, Nikon, Japan).

#### Scratch and tubulogenesis assay

To investigate cellular migratory capabilities post the respective group treatments, we conducted experiments using 6-well plates. Initially, 5 × 10^5^ L929 cells were seeded in each well and cultured for 12 h to allow cell adhesion. When the density of L929 cells reached approximately 90%, a simulated wound was introduced by making a longitudinal incision. Subsequently, PCH-based nanofiber membranes from each group were co-incubated with the cells, and at this time, the NIR exposure groups were subjected to the aforementioned NIR on/off cyclic exposure. The nanofiber membranes were placed in transwell chambers (pore size of 3 μm, CORNING) and were then co-incubated at 37 °C and 5% CO^2^ with the cells, which were not in the transwell chamber. Cellular migration was monitored at 0, 1, and 3 days post co-incubation using an inverted phase-contrast microscope (ECLIPSE Ti2-E, Nikon, Japan).

The capacity of the nanofiber membrane to instigate vascular formation was assessed through a tubulogenesis assay. A 24-well plate, pre-chilled to 4 °C, was laden with 300 μL of Matrigel substrate and subsequently allowed to solidify at 37 °C for 30 min. Human umbilical vein endothelial cells (HUVECs) were prepared at a concentration of 1 × 10^5^ cells/ml in a complete culture medium. Each well received 300 μL of the cell suspension, amounting to approximately 3 × 10^4^ cells. After undergoing respective group treatments, the cells were co-incubated with nanofiber membrane samples from the respective groups and incubated at 37 °C in a 5% CO_2_ environment. Simultaneously, cells were co-incubated under the same conditions with different concentrations of VEGF (0, 25, 75, 100 ng/mL). Following a 4-h incubation, the emergence of tube-like structures was observed and documented using an inverted phase-contrast microscope (ECLIPSE Ti2-E, Nikon, Japan). A quantitative analysis of the acquired image data was performed utilizing ImageJ software (v1.53).

#### Antibacterial assay

Bacterial strains MSSA (ATCC 25923), MRSA (ATCC 43300), and *E. coli* (ATCC 252922) underwent cultivation in Luria–Bertani (LB) broth, utilizing a volume of 4 mL, for a duration of 18 h (1 × 10^8^ CFU/mL). Thereafter, a 48-well plate was prepared with each well containing a 1 mL LB and 20 µL of the respective bacterial culture and incubated at 37 °C for 12 h. Subsequently, either PCH, PCH-GSNO, PCH-PANI, or PCH-PANI-GSNO was introduced for an additional 12-h co-incubation under identical conditions, followed by NIR exposure for the NIR exposure groups to verify the effect bestowed upon the NIR exposure groups by NIR on/off cyclic exposure. The NIR exposure groups were subjected to NIR irradiation at a wavelength of 808 nm and a power density of 0.5 W/cm^2^, in contrast to the non-NIR exposed groups. Each irradiation session lasted for 25 s, followed by a cooling period until the temperature returned to 37 °C before the next cycle, with the total duration being 5 min. The bacterial suspensions, processed as delineated previously, were subsequently employed for the evaluation of the antibacterial properties of various groups.

For live/dead bacterial staining assay, bacterial samples were collected, washed with PBS, and centrifuged for 3 min at 3000 rpm. These samples were then stained with a live/dead bacterial staining kit (Bestbio, China) for 15 min and examined using an inverted fluorescence microscope (ECLIPSE Ti2-E, Nikon, Japan). The data obtained were used to determine the bacterial live ratio using the equation:

5 $${bacterial\,\,live\,\,ratio(\%) = [{N_{live}}/(N{_{live}} + {N_{dead}})] \times 100\% }$$

N_live_ represents the count of live bacteria, while N_dead_ indicates the count of dead ones.

The post-treatment colony-forming units (CFU) were quantified using the standard plate count method. The aforementioned resulting bacterial solutions were diluted to a factor of 5.0 × 10^3^, with 20 µL from each dilution spread onto agar plates. After incubating the agar plates at 37 °C for 24 h, the resulting colony-forming units (CFUs) were counted and assessed using the ImageJ software (v1.53).

#### Antibiofilm assay

For biofilm generation, 500 μL aliquots of MSSA, MRSA, and *E. coli* suspensions (1 × 10^8^ CFU/mL) were separately inoculated into distinct wells of a 48-well plate, followed by a two-day cultivation period with daily replenishment of LB medium. Post-incubation, non-adherent bacteria were removed by aspirating the medium and performing three successive PBS washes. The biofilms were then harvested and subjected to various treatments. Biofilms from both the non-NIR exposed groups and the NIR exposed groups were co-incubated with various PCH-based nanofiber membranes for 12 h. Subsequently, the NIR exposed groups underwent the aforementioned NIR on/off cyclic exposure to verify the enhancement of antibiofilm properties in the PCH-PANI and PCH-PANI-GSNO groups due to NIR exposure. These biofilms were then used for subsequent experimental analyses.

The biofilm mass was quantitatively assessed post-treatment using crystal violet staining. This process involved fixing the biofilm with 200 μL of 4% paraformaldehyde for 15 min, followed by gently washing twice with PBS. Then, the biofilms were stained with 200 μL of 0.1% crystal violet solution for 10 min. After staining, each well was vigorously washed three times with sterile PBS to remove any unbound crystal violet dye. Finally, the biofilms stained with crystal violet were dissolved in 200 μL of 33% acetic acid solution. The quantity of the biofilm was determined by measuring the absorbance of the samples at 590 nm using a microplate reader (SpectraMax i3x, MOLECULAR DEVICES, Germany).

For three-dimensional biofilm structure visualization, biofilms treated were stained using a live/dead bacterial staining kit (Bestbio, China), and observed with an inverted fluorescence microscope (ECLIPSE Ti2-E, Nikon, Japan) after incubating in the dark for one hour. Additionally, the efficacy of biofilm eradication was further validated through scanning electron microscopy (SEM) analysis. Using the same method, biofilms cultivated in 48-well plates with titanium sheets underwent various treatments. Subsequently, the titanium sheets covered with biofilms were fixed in 2.5% glutaraldehyde (GA) at 4 °C for 12 h, followed by dehydration through an ethanol gradient (50%, 60%, 70%, 80%, 90%, and 100%) for 10 min each at 25 °C [32]. After freeze-drying and gold sputtering, SEM (Sigma 300, ZEISS, Germany) was used for observation.

#### Utilizing diabetic Sprague–Dawley rat models

The experimental protocol involving animals was sanctioned by the Institutional Animal Care and Use Committee at Nanfang Hospital, affiliated with Southern Medical University (R202009.05, Guangzhou, China). Each Sprague–Dawley rat was housed individually in cages, with the animal room maintained at a temperature of 25 °C and a relative humidity of 50%, ensuring ventilation and natural light exposure, while also guaranteeing the cleanliness and accessibility of food and water. Prior to experimental treatment, the Sprague–Dawley rats were housed under these conditions for a week to ensure their acclimatization. For the study, 30 male Sprague–Dawley (SD) rats, each weighing approximately 200 g, were procured from the Experimental Animal Center at Southern Medical University. The diabetes model was established in the male SD rats, utilizing methodologies delineated in previous literature [33, 34]. Diabetes was induced by intraperitoneal delivery of streptozotocin (Selleck, USA) with the dosage set at 55 mg per kilogram of body weight. Assessment of blood sugar levels occurred at intervals: days 0, 3, 6, and 7. The Bayer Contour Next EZ instrument (Leverkusen, Germany) facilitated the evaluation of glucose in blood samples drawn from the tail veins of rats. Individuals showcasing glucose values above 11.1 mmol/L on the 6th and 7th days post-injection of streptozotocin were identified as diabetic.

#### Animal procedures and surgeries

The aforementioned 30 diabetic SD rats were subsequently administered an intraperitoneal anesthetic dose of 3% pentobarbital at a volume of 100 μL per 100 g of body weight. After depilation and aseptic preparation, two full-thickness cutaneous defects, each with a diameter of 1.0 cm, were created in symmetrical areas on the back of each rat using a punch device. The wounds were inoculated with a suspension of MRSA, applying 100 μL containing 1 × 10^8^ CFU/mL. To facilitate bacterial colonization, the lesions were occluded with sterile gauze for 8 h. Subsequently, the rats were divided into five groups: control, PCH, PCH-GSNO, PCH-PANI, and PCH-PANI-GSNO, based on the treatment method. Each rat's wounds were divided into two sides; one side received only various dressing treatments, while the other side, in addition to receiving various dressing treatments, also underwent NIR irradiation therapy. The wounds on the sides exposed to NIR underwent NIR on/off cyclic irradiation at a wavelength of 808 nm and a power density of 0.5 W/cm^2^. Each session involved 25 s of irradiation, followed by a cooling period until the temperature returned to 37 °C before commencing the next cycle, with the entire process spanning a total of 5 min. This NIR irradiation treatment was administered to the NIR exposure groups once every two days, totaling seven sessions from day 0 to day 14. The progression of wound healing was systematically documented through photographic records captured on days 0, 1, 3, 7, and 14 post-operative. The housing environment for the SD rats and the method for dressing fixation are shown in Additional file 1: Fig. S3. An Elizabethan collar was used for each SD rat to prevent them from gnawing at the wounds and disrupting the wound dressings.

#### Histological analysis

On days 7 and 14 after the treatments, the SD rats from each group were euthanized (15 SD rats at each time point), and skin tissues from the wounds were subjected to fixation, dehydration, trimming, embedding, sectioning, Hematoxylin and Eosin (H&E) staining, and Masson’s trichrome staining, followed by coverslipping. Observations and image collections of the sections were then conducted using a digital slide scanner. Initially, tissues were captured at 40 × magnification to examine general pathological changes, followed by capturing images at 50 × and 200 × magnification to investigate specific lesion areas. In Masson’s trichrome staining, aniline blue colors collagen fibers blue, while muscle fibers, cellulose, and muscles are stained red by acid fuchsin and picrosirius red; the cytoplasm of red blood cells is stained orange-red, and nuclei are stained black-blue. Appropriate fields of view were selected for the collection of HE and Masson's trichrome staining images, and the length of scar healing, thickness of the epidermal layer, and the number of blood vessels were analyzed using Image J software.

#### Transcriptome sequencing analysis

First, RNA was extracted and purified from L929 cell samples that were subjected to either a control treatment or a treatment with PCH-PANI-GSNO + NIR, with each treatment group consisting of three samples. The extraction and purification were carried out using TRIzol reagent (Invitrogen, CA, USA), in accordance with the manufacturer's instructions. The concentration and purity of the total RNA were then assessed using a NanoDrop ND-1000 spectrophotometer (NanoDrop, Wilmington, DE, USA). The integrity of RNA was evaluated with a Bioanalyzer 2100 (Agilent, CA, USA) and further verified by agarose gel electrophoresis. Samples were considered suitable for subsequent experiments only if the concentration exceeded 50 ng/μL, the RIN value was above 7.0, the OD260/280 ratio surpassed 1.8, and the total RNA amount was over 1 μg. Next, mRNA containing a PolyA tail was specifically captured through two rounds of purification using Dynabeads Oligo (dT) magnetic beads (Catalog #25-61005, Thermo Fisher, USA). The captured mRNA was then fragmented under high temperatures using the NEBNext^®^ Magnesium RNA Fragmentation Module (Catalog #E6150S, USA) for 5–7 min at 94 °C. Subsequently, fragmented RNA was converted to cDNA using Invitrogen SuperScript™ II Reverse Transcriptase (Catalog #1896649, CA, USA). Second-strand DNA synthesis was performed using *E. coli* DNA polymerase I (NEB, Catalog #m0209, USA) and RNase H (NEB, Catalog #m0297, USA), and double-stranded DNA ends were smoothed using dA-tailing Solution (Thermo Fisher, Catalog #R0133, CA, USA). A bases were then added to the blunt ends of the DNA strands, and specific-sized fragments were selected and purified using bead technology. After double-stranded DNA processing with UDG enzyme (NEB, Catalog #m0280, MA, US), PCR amplification was performed with the following program: 95 °C for 3 min pre-denaturation, followed by 8 cycles of 98 °C denaturation for 15 s, annealing at 60 °C for 15 s, and extension at 72 °C for 30 s, with a final extension at 72 °C for 5 min. This process aimed to generate a library with fragment lengths of approximately 300 bp ± 50 bp. Finally, paired-end sequencing was conducted using the Illumina Novaseq™ 6000 sequencer (LC Bio Technology CO., Ltd., Hangzhou, China) with a sequencing mode set to PE150. The raw sequencing data, in fastq format, were initially subjected to quality control using the fastp software (available at https://github.com/OpenGene/fastp), which involved the removal of adapter sequences, duplicate sequences, and low-quality sequences using the software's default settings. The data were then mapped to the human genome GRCh38 using the HISAT2 tool (details at https://ccb.jhu.edu/software/hisat2), with the mapped data saved in bam format. Gene and transcript assembly were performed using StringTie software, and quantification analysis was conducted using the FPKM method, with the formula FPKM = total exon fragments / mapped reads (in millions) × exon length (in kilobases). Differential expression analysis was completed using the edgeR package (refer to https://bioconductor.org/packages/release/bioc/html/edgeR.html), setting the criteria for differentially expressed genes as a fold change greater than 2 or less than 0.5, and a p-value less than 0.05. Finally, the identified genes were subjected to Gene Ontology (GO) and Kyoto Encyclopedia of Genes and Genomes (KEGG) enrichment analysis using the DAVID tool (available at https://david.ncifcrf.gov/).

#### Statistical analysis

Statistical analyses of the experimental data were conducted using GraphPad Prism 8.0 software. We employed one-way analysis of variance (ANOVA), followed by Bonferroni’s post hoc test, to discern statistical significance. The error bars illustrated in the figures represent the mean ± standard deviation (SD). Levels of significance are denoted as follows: ∗P < 0.05, ∗∗P < 0.01, ∗∗∗P < 0.001, and ∗∗∗∗P < 0.0001.

## Results

### Fabrication and characterization of nanofibers

#### SEM scan of the nanofiber membranes

**Fig. 2:**
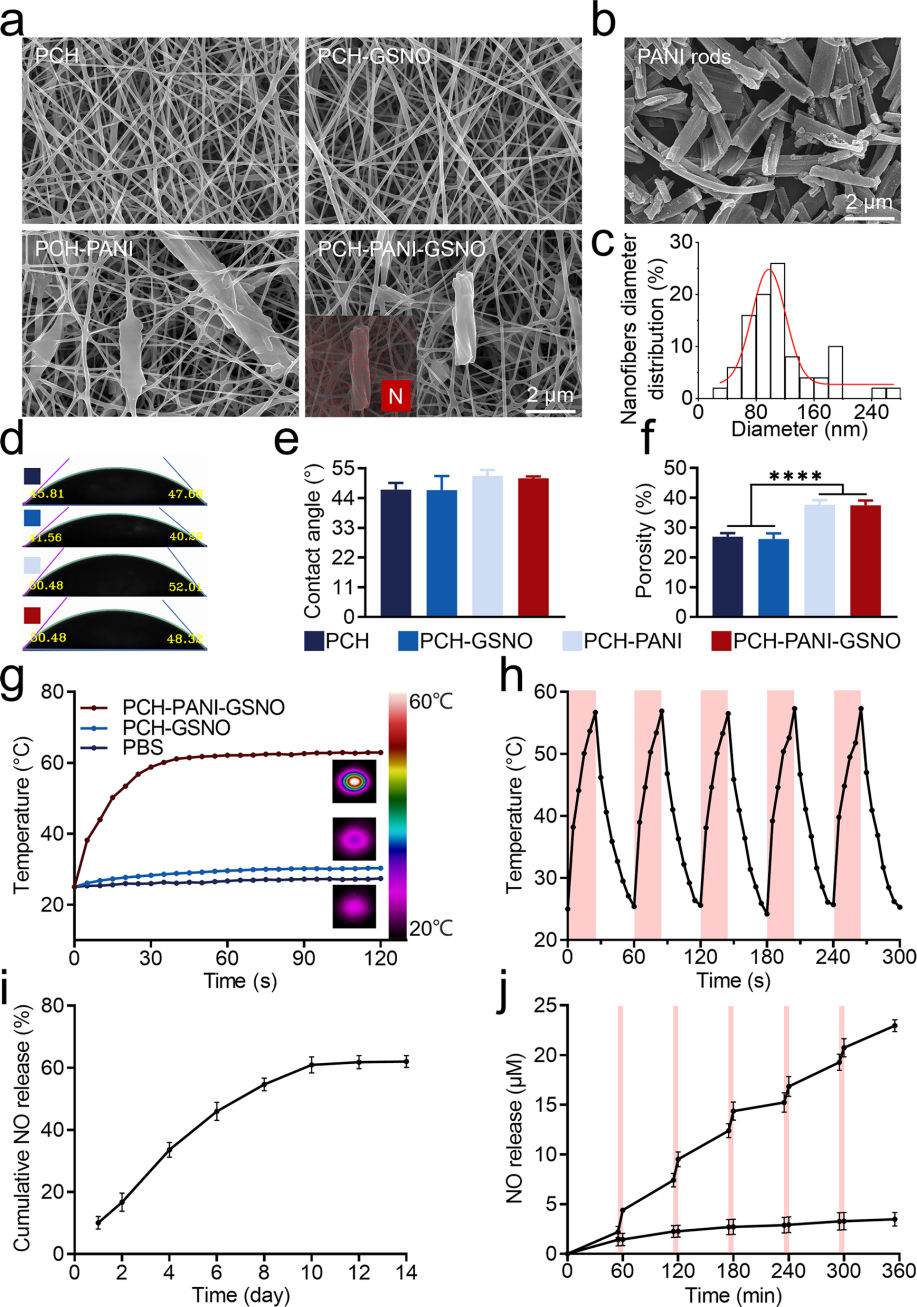
Characterization of PCH-based nanofiber membranes. **a** The SEM image of the PCH, PCH-GSNO, PCH-PANI, and PCH-PANI-GSNO nanofiber membranes; including N-element mapping scan of PCH-PANI-GSNO nanofiber membrane. **b** The SEM images of PANI rods. **c** The diameter distribution of the PCH-PANI-GSNO nanofibers. **d**, **e** The contact angle of the PCH, PCH-GSNO, PCH-PANI, and PCH-PANI-GSNO nanofiber membranes. **f** Porosity of the PCH, PCH-GSNO, PCH-PANI, and PCH-PANI-GSNO nanofiber membranes. **g** Under NIR exposure (808 nm, 0.5 W/cm^2^), the temperature variation curves for PCH-PANI-GSNO, PCH-GSNO, and PBS. **h** Heating and cooling cycles of PCH-PANI-GSNO under five rounds of NIR exposure (808 nm, 0.5 W/cm^2^) in a photothermal context. **i** The cumulative NO release profiles of the PCH-PANI-GSNO were measured on days 1, 2, 4, 6, 8, 10, 12, and 14. **j** Under NIR exposure (808 nm, 0.5 W/cm^2^), the on/off NO release profile (the top curve) of PCH-PANI-GSNO and the NO release profile (the bottom curve) of PCH-GSNO (n = 3, ∗*P* < 0.05, ∗∗*P* < 0.01, ∗∗∗*P* < 0.001, ∗∗∗∗*P* < 0.0001)

In our attempts, when the ratio of PVA to CS was set at 50%:50% (w/w) and 60%:40% (w/w), it was impossible to electrospin smooth, uniform, and bead-free nanofiber membranes. This indicates that a disproportionately high proportion of CS hampers the electrospinning process, leading to its failure. At a PVA:CS ratio of 70%:30% (w/w), although we successfully spun nanofiber membranes, the morphology of the electrospun fibers, as observed under SEM (Additional file 1: Fig. S4a), was unacceptable due to the bead-on-string structure. However, at a PVA:CS ratio of 80%:20% (w/w), the SEM images of the nanofibers exhibited a satisfactory morphology. Moreover, the introduction of HTCC, PANI, and GSNO did not cause significant morphological disruption. Additional file 1: Fig. S2 illustrates the methodology for creating PCH-based nanofiber membranes. SEM images of these membranes and PANI rods are presented in Fig. 2a, b. The PANI rods, characterized by a major axis length of 2.06 ± 0.80 μm and a minor axis length of 0.47 ± 0.17 μm, are uniformly dispersed amidst the fibers. Dimensional analyses of the PANI rods are depicted in Additional file 1: Figs. S4b-c. Figure 2c and Additional file 1: Figs. S4d-f provide an in-depth visualization of the nanofibers, which PCH-PANI-GSNO nanofibers exhibit an average diameter of 114.55 ± 49.03 nm. Significantly, Fig. 2a presents the elemental mapping scan of nitrogen within the PCH-PANI-GSNO nanofiber membranes, indicating the uniform distribution of GSNO within the PCH-PANI-GSNO nanofiber membranes. Utilizing SEM images, we assessed the porosity of PCH-based nanofiber membranes using ImageJ software (v1.53). As observed in Fig. 2f, the introduction of PANI rods results in finer diameters of the nanofibers (Fig. 2c and Additional file 1: Figs. S4d-f), which in turn leads to a higher porosity rate.

#### Spectral analysis via FT-IR

As presented in Additional file 1: Fig. S5, due to PVA being the predominant component in PCH-based nanofiber membranes, its functional group absorption is significantly stronger compared to other constituents, endowing the PCH-based nanofiber membranes with distinct PVA characteristics. The composite membrane itself does not exhibit new characteristic peaks, indicating the absence of novel chemical bond formation. However, the shift of the PVA 3422 cm^−1^ OH group absorption towards lower wavenumbers suggests an overall reduction in energy. This is presumably due to hydrogen bonding between un-crosslinked segments of CS and PVA, which enhances intermolecular forces and results in more extensive crosslinking. Among the four composite membranes, the proportion of PANI is too small to significantly impact the PVA-CS-HTCC-PANI sample, leading to absorption intensities almost identical to those of PVA-CS-HTCC. In contrast, PVA-CS-HTCC-GSNO shows stronger absorption than PVA-CS-HTCC, likely due to the addition of GSNO altering the intrinsic refractive index of the sample, thereby increasing it. In the ATR mode, as the refractive index of each band increases, the penetration depth also increases, leading to reduced reflection and enhanced infrared absorption. The weakest IR absorption is observed in PVA-CS-HTCC-PANI-GSNO, potentially due to the combined interactions of the five components, which alter the structure of the composite membrane, resulting in the lowest refractive index. This causes a reduction in absorption across various bands due to decreased penetration depth, under the premise that the functional groups remain essentially unchanged.

#### Thermogravimetric analysis and contact angle analysis

The thermogravimetric analysis (TGA) results for the PCH-based nanofiber membranes are delineated in Additional file 1: Fig. S6a. The TGA curve of the PCH-PANI-GSNO nanofiber membrane is principally segmented into four distinct thermal decomposition phases. The most pronounced mass decrement is evident in the second phase, which extends from 159.38 °C to 371.30 °C. Importantly, this interval exceeds the temperature necessary for therapeutic applications, underscoring the nanofiber membrane's capacity to maintain its chemical and physical integrity under therapeutic thermal conditions. The optical water contact angle measurements for the nanofiber membranes, specifically PCH, PCH-GSNO, PCH-PANI, and PCH-PANI-GSNO, are presented in Fig. 2d, e. The water contact angle of the PCH-PANI-GSNO nanofiber membrane is 51.28 ± 0.69°. Comparative analysis across the variants revealed no statistically significant differences, signifying that the incorporation of PANI and GSNO does not alter this property. These measurements underscore the intrinsic hydrophilicity characteristic of PCH-based nanofiber membranes, essential for wound dressings.

#### Water absorption and degradation rate assessment of nanofiber membranes

Additional file 1: Fig. S6b delineates the dynamic water absorption ratios of the nanofiber membranes across the quartet of sample groups. The water absorption ratios of PCH-based nanofiber membranes rapidly increase within the initial 12 h and tend to stabilize at 72 h. The water absorption ratios for PCH, PCH-GSNO, PCH-PANI, and PCH-PANI-GSNO are 291.67 ± 24.66%, 289.33 ± 18.01%, 300.00 ± 5.00%, and 300.00 ± 13.23%, respectively. Furthermore, under NIR irradiation, the changes in water absorption ratios of PCH-PANI-GSNO + NIR showed no statistical difference when compared with the other groups (305 ± 13.45%). Upon immersion in PBS, each nanofiber membrane manifested an absorption capacity ranging from 275 to 320% of its respective weight. Additional file 1: Fig. S6c suggests that the investigation into the degradation kinetics of the nanofiber membrane assemblies over a 14-day period demonstrated an initial pronounced diminution in mass, with a reduction to 75–78% of the baseline on the initial day. This phenomenon is attributed primarily to the liberation of incorporated agents such as CS and HTCC. Subsequent to this initial phase, the degradation rate decelerated, culminating in a relative stabilization at approximately 30–35% of the original mass by the fourteenth day. After introducing on/off cycles of NIR irradiation, it can be observed that the degradation rate of the PCH-PANI-GSNO nanofiber membranes is faster compared to those of the other groups, ultimately stabilizing at around 20% of the original mass by day 14.

In the degradation rate assessment experiment, we conducted SEM observations of the PCH-PANI-GSNO nanofiber membranes on day 3, day 7, and day 14 (Additional file 1: Fig. S7). Over time (alongside drug release and NIR irradiation), the nanofiber membranes maintained a stable, loose, mesh-like skeletal structure. It was also observed that the nanofibers within the membranes gradually became finer during the degradation process.

#### Photothermal activity of nanofiber membranes

Additional file 1: Fig. S6d displays the temperature change curves of the PCH-PANI-GSNO nanofiber membrane under NIR irradiation at different power densities. It is observable that at an NIR power density of 0.5 W/cm^2^, the temperature can reach approximately 60 °C after 30 s. However, when the NIR power density is increased to 1 W/cm^2^, the temperature change of the PCH-PANI-GSNO nanofiber membrane is very dramatic, soaring to around 120 °C within 30 s, with a continuing upward trend. Considering such high temperatures and the difficult-to-control rate of temperature change may cause damage to the wound, we selected an NIR power density of 0.5 W/cm^2^.

Upon exposure to NIR irradiation, the photothermal properties of the nanofiber membranes were scrutinized through the observation of temperature fluctuations. The PCH-PANI-GSNO membranes exhibited an expeditious elevation in surface temperature, reaching approximately 58 °C within a 30-s duration and subsequently attaining a stable plateau at an approximate temperature of 62 °C, as illustrated in Fig. 2g. Conversely, the PCH-GSNO membranes, under identical irradiation conditions, only managed to attain a surface temperature of 30.3 °C within a span of 2 min, while the temperature variation in PBS remained inconsequential. The photothermal resilience of PCH-PANI-GSNO was further evaluated. Figure 2h delineates that through five cycles of laser activation and deactivation, it was observed that the temperature of nanofiber membrane responded rapidly and sensitively to changes in NIR irradiation, without significant decay in temperature increase. This indicates that PCH-PANI-GSNO demonstrates considerable tolerance to sustained laser exposure, exhibiting notable photothermal stability.

#### NO release and NIR-responsive NO burst from PCH-PANI-GSNO

Employing the Griess assay, a consistent release of NO at ambient temperature for a duration spanning 10 to 12 days was observed in the PCH-PANI-GSNO nanofiber membrane, suffused with GSNO. This timeframe aligns with the principal wound healing period observed in the PCH-PANI-GSNO group in subsequent animal studies, as indicated in Fig. 2i. Crucially, considering the photosensitive and thermosensitive characteristics of GSNO, the PCH-GSNO-PANI nanofiber membrane demonstrates a nanodetonator-like, controlled, and rapid NO release, proportional to the duration of NIR laser irradiation exposure (Fig. 2j).

### Cell viability and biocompatibility assay

The loading contents of HTCC, PANI, and GSNO were determined through the Cell viability assay, under the premise of ensuring the electrospinnability of the polymer (refer to the Additional file 1). CCK-8 assay outcomes (Additional file 1: Fig. S8a) revealed that the viability of L929 cells remained unimpaired (< 80%) until the concentration of HTCC reached 5% w/w. For PANI and GSNO, the respective concentration thresholds were identified as 5% w/w and 15% w/w. Importantly, the simultaneous application of these substances at the stipulated concentrations did not elicit cytotoxic effects (Additional file 1: Fig. S8b).

The viability of L929 cells under treatment was assessed using a live/dead cell staining assay, with the results showing high cell viability (> 95%) in all groups on both day 1 and day 3. (Additional file 1: Fig. S9). During this period, the cell count in all samples demonstrated an average increase of 2.26 to 2.86 times, indicating that the PCH-based nanofiber membranes did not exhibit significant toxicity to cell growth. The viability was further evaluated by analyzing the cell-spreading area, aligning with methodologies referenced in previous studies [35]. Additional file 1: Fig. S10 corroborates that in each treatment group, the spreading area of L929 cells on average increased to 1.99–2.35 times the original size within three days, indicating that the PCH-based nanofiber membranes did not significantly impede cell growth. The staining results were corroborated by the lack of notable differences in the area covered by cell spreading across the groups.

### Intracellular NO detection, scratch and tubulogenesis assay

**Fig. 3:**
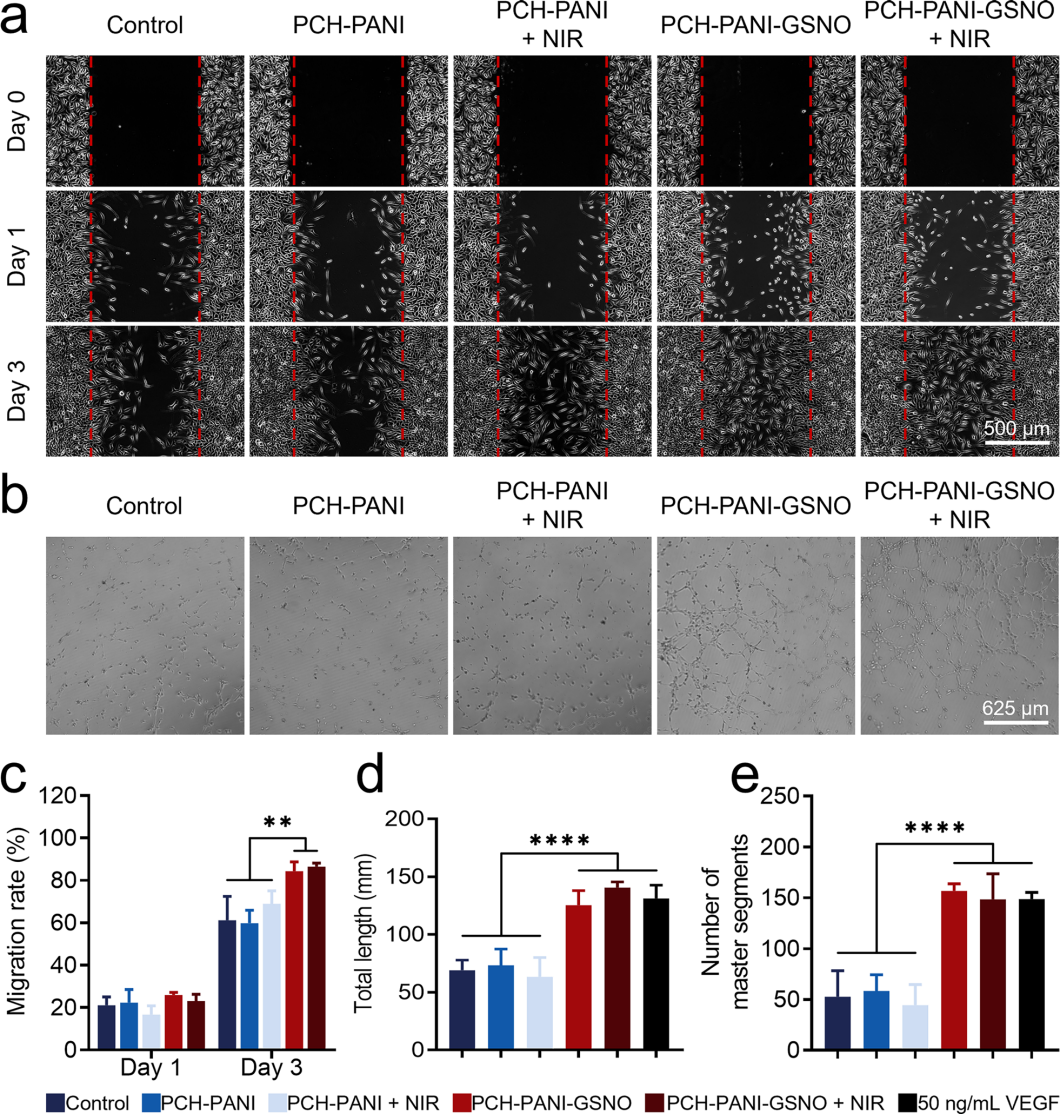
Analysis of the promotion of cell migration and angiogenic properties of the PCH-based nanofiber membrane. **a**, **c** The scratch assay outcomes and analysis of the migration rate for L929 cells post-treatment with PCH-based nanofiber membranes on days 0, 1, and 3. **b**, **d**, **e** The tube-forming ability of HUVECs after treatment with PCH-based nanofiber membranes. (Three biological replicates; images from three fields of view each; ∗*P* < 0.05, ∗∗*P* < 0.01, ∗∗∗*P* < 0.001, ∗∗∗∗*P* < 0.0001)

In accordance with the data delineated in Additional file 1: Fig. S11, the incorporation of GSNO into nanofiber membranes (PCH-GSNO ± NIR and PCH-PANI-GSNO ± NIR groups) engendered the perceptible generation of intracellular NO subsequent to cellular co-incubate. This observation substantiates the efficacious translocation of NO, liberated from the nanofiber matrices, into the intracellular milieu. During the cell scratch assay (Fig. 3a, c and Additional file 1: Figs. S12a, d), a temporal progression in the migration of L929 cells towards the void region was discerned. By day three, the cohorts subjected to PCH-GSNO, PCH-GSNO + NIR, PCH-PANI-GSNO, and PCH-PANI-GSNO + NIR manifested augmented migration rates (86.21 ± 3.13%, 85.12 ± 4.87%,84.30 ± 4.38%, 86.50 ± 1.67%, respectively) relative to the control and control + NIR groups (61.18 ± 11.38%, 56.54 ± 13.95%). This occurrence is ascribed to the modulated liberation of NO from the nanofiber membranes, facilitating L929 cell motility and concomitantly augmenting wound healing. Assessment of tube formation enabled the quantification of total vessel length and master segment count. In terms of the total length in HUVECs tubulogenesis, compared to the control and control + NIR groups’ 69.05 ± 8.79 mm and 63.60 ± 11.95 mm, the total lengths following treatment with PCH-GSNO, PCH-GSNO + NIR, PCH-PANI-GSNO, and PCH-PANI-GSNO + NIR were increased to 131.38 ± 3.34 mm, 120.31 ± 10.00 mm, 125.31 ± 12.61 mm, and 140.64 ± 4.91 mm, respectively. These data are equivalent to 50 ng/mL of VEGF (131.221 ± 11.52 mm, no statistical difference). Regarding the number of master segments in tubulogenesis, compared to the control and control + NIR groups’ 52.67 ± 25.58 and 46.33 ± 14.05, the numbers following treatment with PCH-GSNO, PCH-GSNO + NIR, PCH-PANI-GSNO, and PCH-PANI-GSNO + NIR were elevated to 153.00 ± 16.64, 156.01 ± 4.58, 156.67 ± 7.09, and 148.33 ± 25.38, respectively. These data are also equivalent to 50 ng/mL of VEGF (148.67 ± 6.66 mm, no statistical difference), indicating that the GSNO-loaded PCH-based nanofiber membranes have a tube-forming capability on HUVECs equivalent to that of 50 ng/mL of VEGF (Fig. 3b, d, e; Additional file 1: Figs. S12b-c, e–f). Relative to both the control and membranes devoid of GSNO, the GSNO-imbued nanofiber membranes conspicuously amplified HUVEC tube genesis.

### Antimicrobial assessment of nanofiber membranes in vitro

**Fig. 4:**
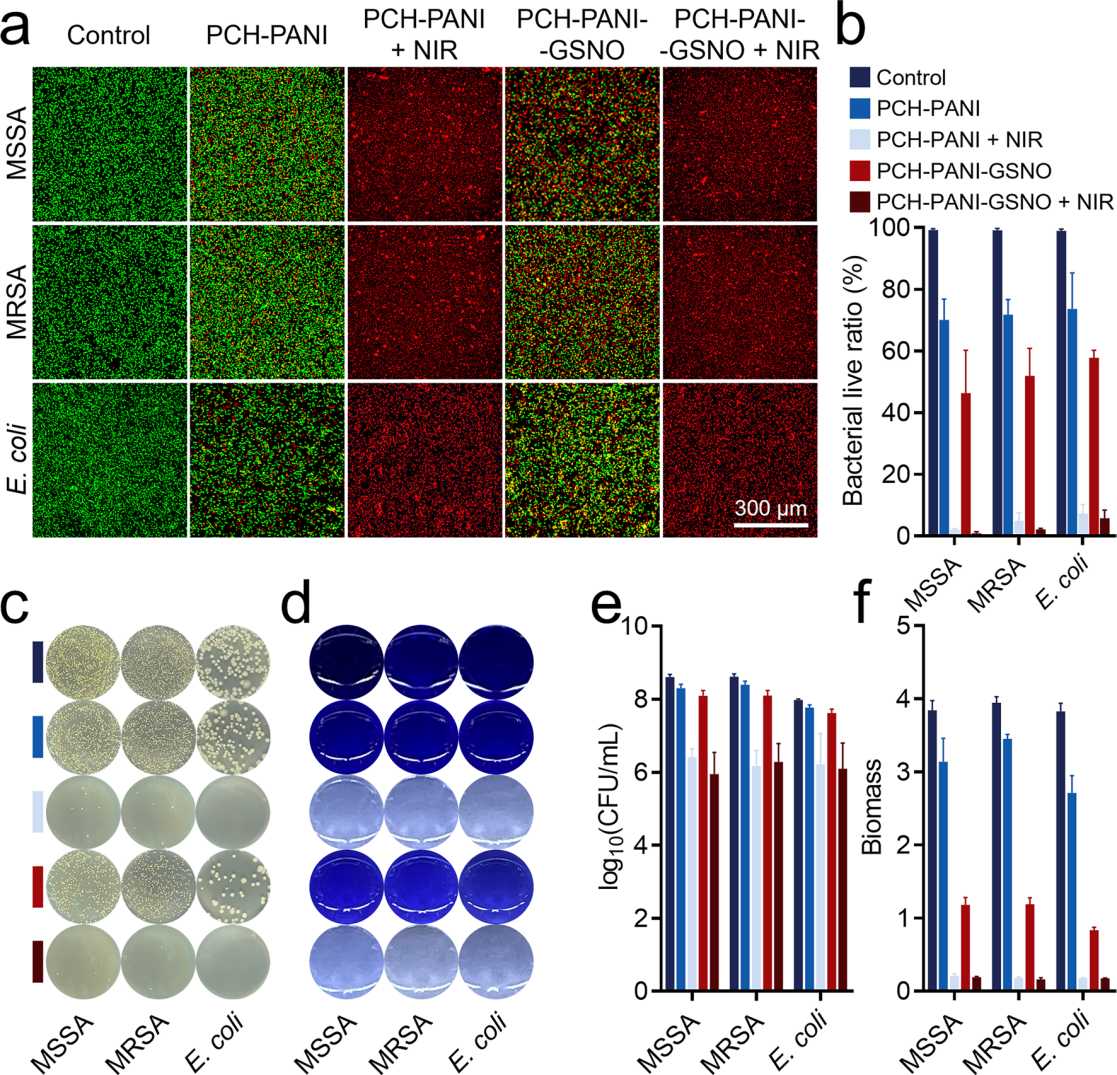
Evaluation of the antibacterial properties of PCH-based nanofibrous membranes in vitro. **a**, **b** Bacterial live/dead staining of MSSA, MRSA and *E. coli* post-treatment with PCH-based nanofiber membranes and subsequent live bacteria ratio quantification. **c**, **e** The CFU counting method tests the in vitro antibacterial effects of PCH-based nanofiber membranes and the quantitative analysis of CFU. **d**, **f** Biofilm formation by MSSA, MRSA, and *E. coli* visualized using crystal violet staining post diverse treatments, and the OD590 value of the biofilms from the three bacteria following crystal violet staining. (Three biological replicates; images from three fields of view each; ∗*P* < 0.05, ∗∗*P* < 0.01, ∗∗∗*P* < 0.001, ∗∗∗∗*P* < 0.0001)

Figure 4a, b and Additional file 1: Figs. S13a-b depict the survival of three bacterial strains post-treatment by various groups, as determined through live/dead bacterial staining assays. Compared to the control and control + NIR groups, a significant reduction in bacterial viability was observed in strains treated with PCH-based nanofiber membranes. The data suggest that PCH group nanofiber membranes maintain their antibacterial activity even without NIR stimulation. The incorporation of the NO donor GSNO endowed the PCH-based nanofiber membranes with NO antibacterial capabilities, enhancing the antibacterial effect in the PCH-GSNO and PCH-PANI-GSNO groups. More notably, upon NIR irradiation of the PCH-PANI and PCH-PANI-GSNO groups, the potent PTT antibacterial ability led to a drastic reduction in the survival rates of all three bacterial strains in the PCH-PANI + NIR and PCH-PANI-GSNO + NIR groups. The survival rates of MSSA, MRSA, and *E. coli* in the PCH-PANI + NIR group decreased to 2.03 ± 0.38, 4.92 ± 2.61, and 7.25 ± 2.97, respectively, while in the PCH-PANI-GSNO + NIR group, they further reduced to 0.88 ± 0.55, 2.19 ± 0.30, and 5.81 ± 2.65. Consistent with the trends corroborated by the live/dead bacterial staining assays, colony count analyses also confirm that the synergistic combination of NO and PTT therapies significantly enhances the antibacterial efficacy of PCH-based nanofiber membranes across various treatment groups. In colony count data for MSSA, MRSA, and *E. coli*, the counts in the control and control + NIR groups were 8.61 ± 0.07, 8.62 ± 0.08, 7.98 ± 0.03 log_10_ (CFU/mL) and 8.61 ± 0.07, 8.62 ± 0.08, 7.98 ± 0.03 log_10_ (CFU/mL) respectively, whereas in the PCH-PANI-GSNO + NIR group, they were 5.95 ± 0.59, 6.28 ± 0.51, and 6.10 ± 0.70 log_10_ (CFU/mL) respectively. This indicates that after treatment with PCH-PANI-GSNO + NIR, the bacterial counts for these three strains decreased by approximately 100 to 1000 times compared to the control group (Fig. 4c, e; Additional file 1: Figs. S13c, e).

### Antibiofilm properties of nanofiber membranes

**Fig. 5:**
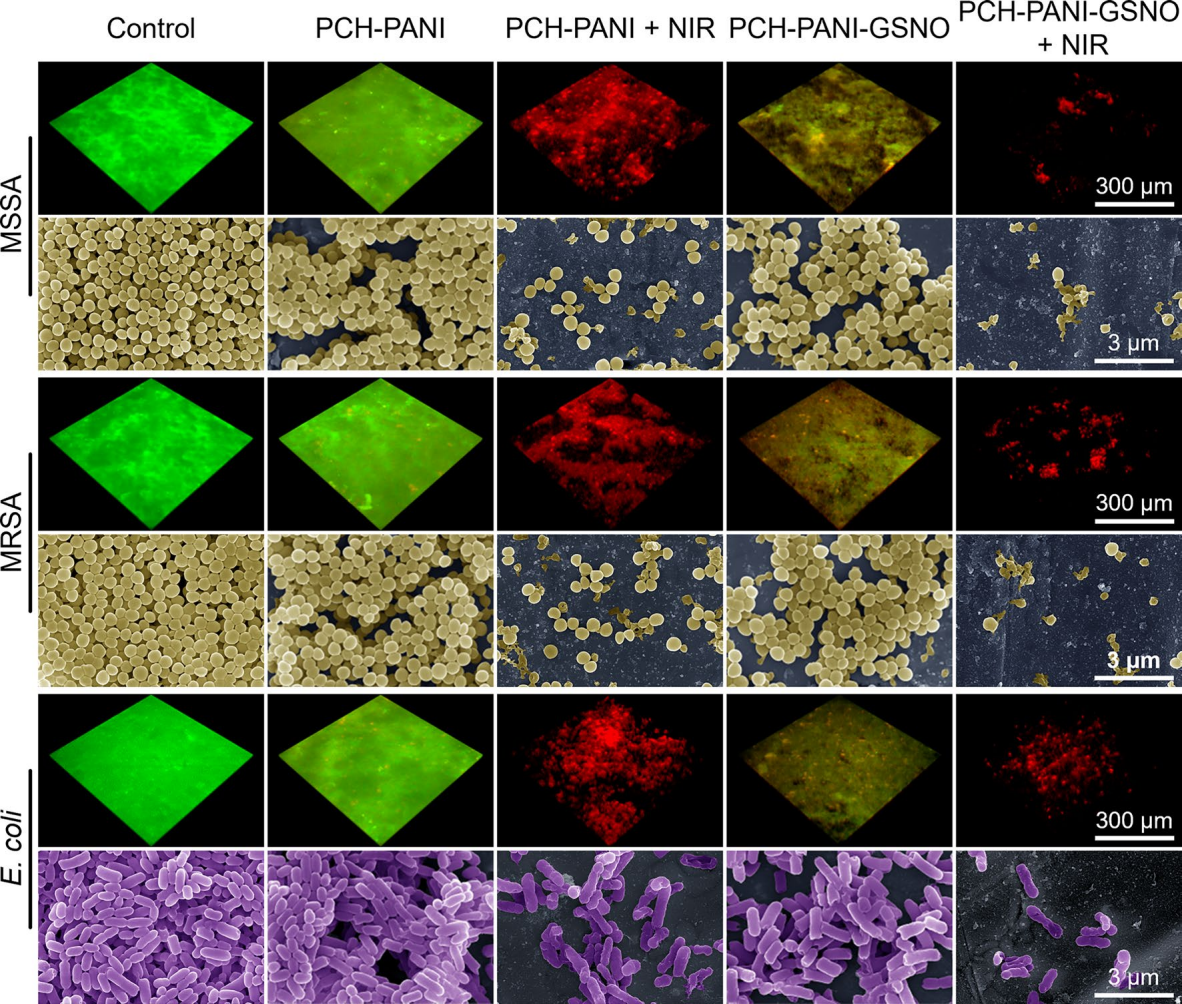
Biofilms response to control, PCH-PANI, PCH-PANI + NIR, PCH-PANI-GSNO, PCH-PANI-GSNO + NIR treatment in SEM and Z-stack scanning. Z-stack scanning from live/dead staining and SEM images of biofilms produced by three types of bacteria (MSSA, MRSA, and *E. coli*) after treatment with PCH-based nanofiber membranes

Analysis of crystal violet staining revealed a reduction in biofilm biomass across all specimens subjected to PCH-based nanofiber membranes. This decrement was accentuated upon the incorporation of NO and NIR irradiation. The absorbance corresponding to the biofilms' biomass of MSSA, MRSA, and *E. coli* in the control and control + NIR groups were 3.84 ± 0.13, 3.95 ± 0.09, 3.83 ± 0.11 and 3.80 ± 0.10, 3.90 ± 0.01, 3.84 ± 0.10 respectively. In the PCH-PANI-GSNO + NIR group, these values were significantly reduced to 0.19 ± 0.01, 0.16 ± 0.02, and 0.18 ± 0.00, respectively (Fig. 4d, f; Additional file 1: Figs. S13d, f). Employing Z-stack scanning through fluorescence microscopy and SEM, it was ascertained that PCH nanofiber membranes efficaciously impeded bacterial proliferation. Subsequent to NO incorporation, there was a discernible dispersion of the biofilm accompanied by a significant increment in deceased bacterial enumerations. In the absence of NIR irradiation, PCH-PANI nanofiber membranes demonstrated biofilm disruption analogous to the PCH group. Under NIR irradiation conditions, although the biofilm was partially preserved, a preponderance of the encapsulated bacteria was determined to be non-viable. Notably, PCH-PANI-GSNO nanofiber membranes, while retaining the biofilm-dispersing properties of the PCH-GSNO group, elicited further disintegration of the biofilm structure upon NIR exposure. A substantial proportion of the bacteria manifested a wrinkled morphology, highlighting the formidable anti-biofilm efficacy achieved by the amalgamation of NO and photothermal therapy (Fig. 5; Additional file 1: Fig. S14).

### Wound healing and antimicrobials assessment in vivo

**Fig. 6:**
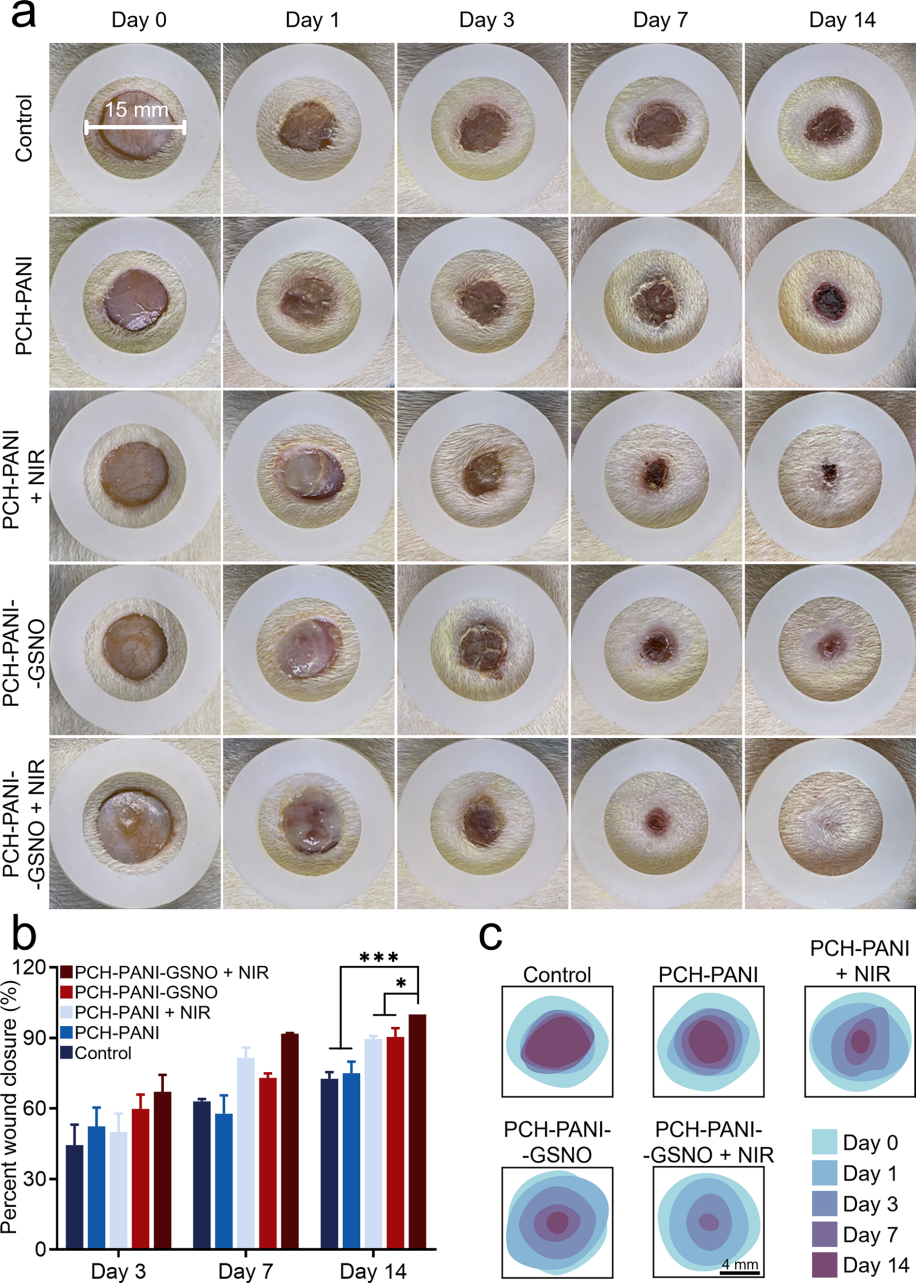
PCH-based nanofiber membranes promoted infected diabetic wound healing. **a** Images depicting wound contraction dynamics on the wound site for the control, PCH-PANI, PCH-PANI + NIR, PCH-PANI-GSNO, and PCH-PANI-GSNO + NIR groups on days 0, 1, 3, 7, and 14. **b** Quantitative analysis of the wound healing rate on days 3, 7, and 14. **c** Simulation of wound dynamics over the 14-day treatment in the control, PCH-PANI, PCH-PANI + NIR, PCH-PANI-GSNO, and PCH-PANI-GSNO + NIR groups. (n = 3; ∗*P* < 0.05, ∗∗*P* < 0.01, ∗∗∗*P* < 0.001, ∗∗∗∗*P* < 0.0001)

This study sought to assess the effectiveness of dressings crafted from electrospun nanofibers in promoting the healing of infected DW in a rat model. Various dressings and groups treated with NIR on/off cyclic irradiation of these nanofiber membranes, were applied to these wounds, as depicted in Fig. 6a and Additional file 1: Fig. S15a. The trajectory of wound healing was tracked using photographic records, with the wound surface area being quantified relative to a reference circular shape made of plastic, 15 mm in diameter. Notably, the PCH-PANI-GSNO + NIR group demonstrated superior healing efficacy among all experimental groups. An exhaustive analysis delineating skin impairments and regenerative trends is presented in Fig. 6b, c and Additional file 1: Figs. S15b-c. Subsequent to a two-week post-surgical period, the rats were subjected to euthanasia for tissue harvesting.

As seen in Additional file 1: Fig. S16, the bacterial load in the wounds of rats from the PCH-PANI-GSNO + NIR group was significantly lower than that of the other groups. On the 3rd day, the bacterial load in the wounds of the PCH-PANI-GSNO + NIR group was about 10% of that in the control group. By the 7th day, over 99% of the bacteria had been cleared in the PCH-PANI-GSNO + NIR group, and the bacterial load was four orders of magnitude lower than that in the PCH-PANI + NIR group.

### Histological analysis

**Fig. 7:**
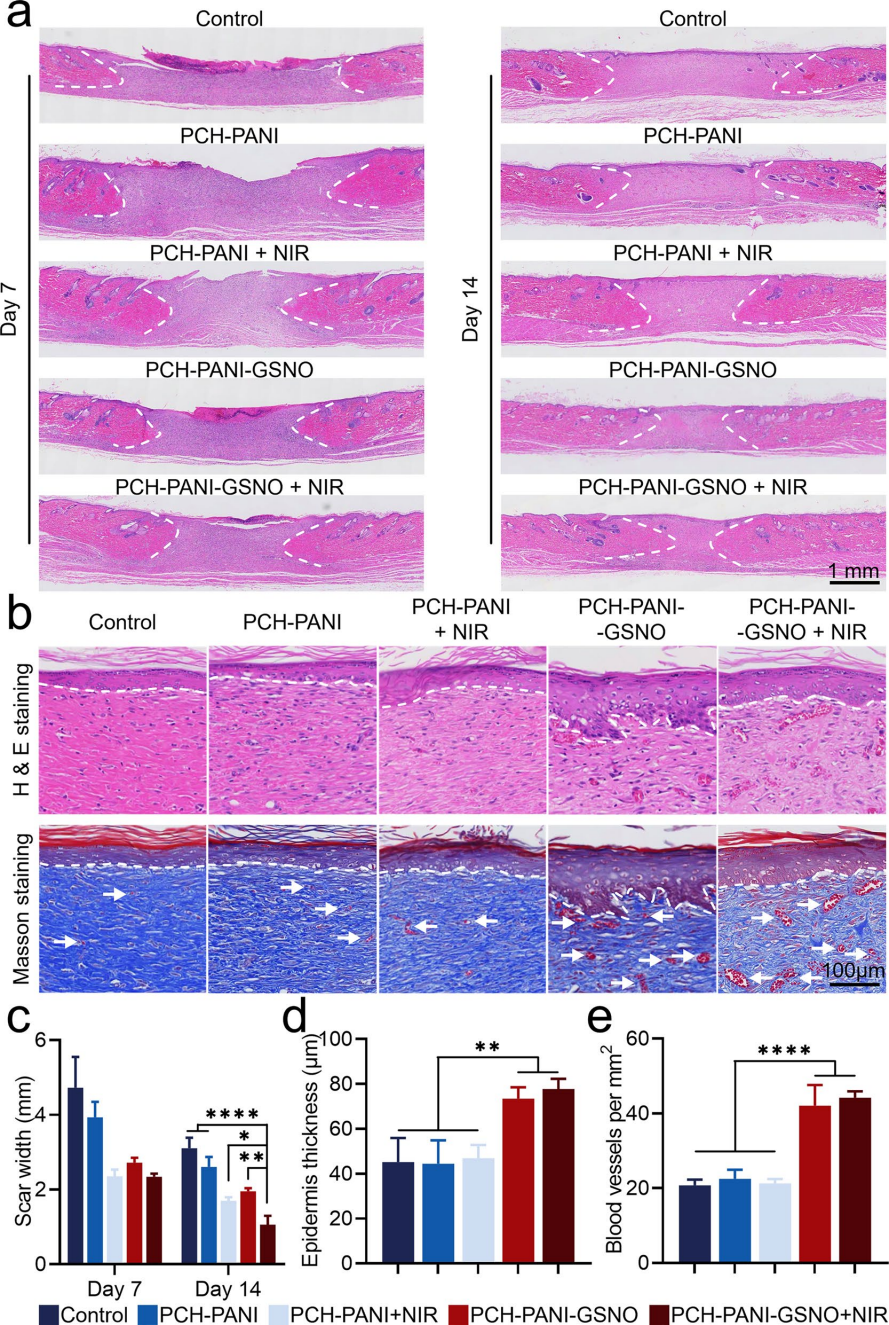
Assessment of wound repair via H&E and Masson staining. **a**, **c** Results of healing wounds on days 7 and 14, represented by H&E staining. White dashed lines mark unhealed scars. Scar widths for various groups analyzed with ImageJ (v1.53) **b** Day 14 skin samples’ H&E and Masson-stained images. Dashed lines denote the epidermis, and arrows point to blood vessels. **d** Epidermal thickness analysis of wounds after nanofiber membrane treatment using ImageJ on day 14 skin samples. **e** Quantification of blood vessels at wound locations via ImageJ on day 14 skin samples. (n = 3; ∗*P* < 0.05, ∗∗*P* < 0.01, ∗∗∗*P* < 0.001, ∗∗∗∗*P* < 0.0001)

The therapeutic potency of the PCH-based nanofiber membrane was evaluated via histological analyses employing Hematoxylin & Eosin (H&E) and Masson’s trichrome staining at distinct time points, namely day 7 and day 14. Figure 7 and Additional file 1: Fig. S17 reveal that the cohort treated with PCH-PANI-GSNO + NIR, which underwent concomitant NO and PTT, exhibited expedited wound recuperation. This was succeeded by the PCH-PANI + NIR cohort and subsequently by the PCH-PANI-GSNO cohort. The scar breadth in subjects administered PCH-PANI-GSNO + NIR manifested a conspicuous contraction compared to other cohorts. Supplementary assessment of cutaneous wound healing quality was conducted utilizing H&E and Masson's trichrome staining methodologies. Data derived from H&E staining underscore that wounds subjected to PCH-PANI-GSNO + NIR treatment, amalgamating both NO and PTT interventions, displayed the most attenuated scar breadth (Fig. 7a, c; Additional file 1: Figs. 17a, c). Masson’s trichrome staining accentuated that, in comparison to alternative cohorts, the epidermal thickness in wounds treated with PCH-PANI-GSNO + NIR was appreciably augmented (Fig. 7b, d; Additional file 1: Figs. 17b, d). Moreover, a marked proliferation in angiogenesis was discerned in the rejuvenated cutaneous tissues of both the PCH-GSNO ± NIR and PCH-PANI-GSNO ± NIR cohorts (Fig. 7b, e; Additional file 1: Figs. 17b, e). In summation, these findings, conjoined with in vitro evidence of angiogenesis, enhanced cellular motility, and anti-infective attributes, advocate for the preeminence of PCH-PANI-GSNO + NIR in facilitating functional skin regeneration.

### Transcriptome sequencing analysis

**Fig. 8:**
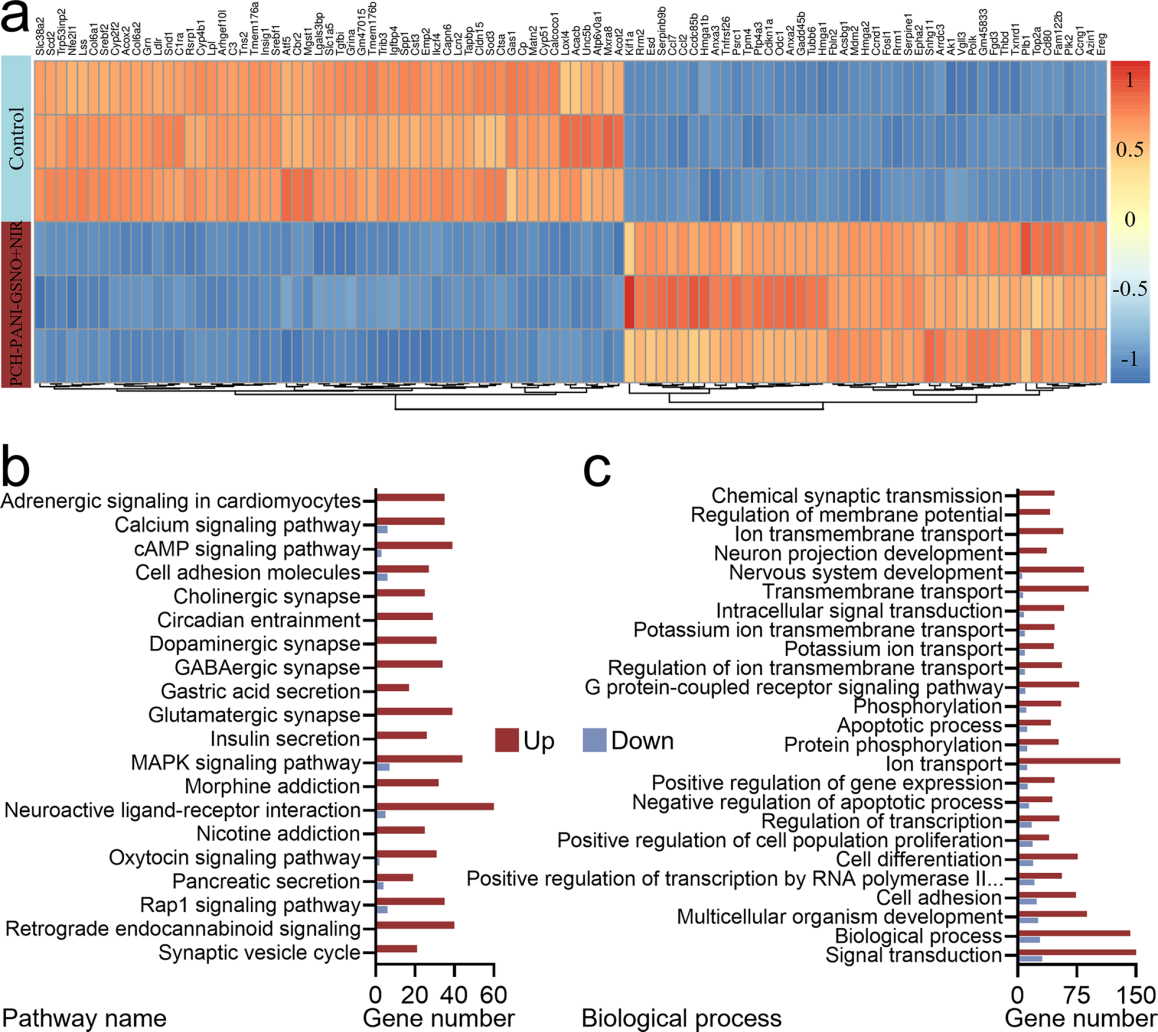
Investigation of the mechanism of PCH-PANI-GSNO + NIR treatment. **a** Heatmap showcasing DEGs between the PCH-PANI-GSNO + NIR treatment and control groups. **b** KEGG pathway enrichment results of the DEGs, presenting the 20 most prominent pathways with both upregulated and downregulated activities. **c** Top 25 biological processes from the GO analysis, highlighting the variance in upregulated and downregulated activities

Transcriptomic analysis via RNA-sequencing was utilized to scrutinize the molecular responses in L929 cells exposed to interventions from both the PCH-PANI-GSNO + NIR and Control cohorts. Additional file 1: Fig. S18 delineates the identification of 1861 differentially expressed genes (DEGs), comprising 1448 up-regulated and 413 down-regulated genes. Figure 8a presents a heatmap illustrating the most significantly up-regulated and down-regulated DEGs. To elucidate the functional implications of these DEGs, analyses were conducted utilizing the Kyoto Encyclopedia of Genes and Genomes (KEGG) pathways and Gene Ontology (GO) terms. Figure 8b underscores salient pathways including “MAPK”, “cAMP”, “Calcium”, “Cell adhesion molecules”, and “Rap1”, which emerged prominently among the top 20 enriched pathways in the KEGG analysis. Concurrently, the GO enrichment analysis underscored the pivotal role of the DEGs in biological processes such as “Cell adhesion”, “Differentiation”, “Regulation of proliferation”, “Regulation of apoptosis”, “Phosphorylation”, “G protein-coupled receptor signaling”, and “Intracellular signaling” (Fig. 8c). In summation, the NO gas-mediated therapy facilitated by the PCH-PANI-GSNO nanofiber membrane adeptly modulated diabetic wound healing processes through the orchestration of pivotal cellular pathways and gene expression profiles, thereby ensuring cellular equilibrium during regenerative phases.

## Discussion

Diabetic wound healing is impeded by the confluence of bacterial infection and compromised perfusion [36]. Extensive research has been devoted to the development of efficacious wound dressings tailored to expedite the recuperation of DW [37]. This investigation successfully synthesized a nanofiber membrane dressing, amalgamating PANI and GSNO, through electrospinning techniques. The resultant dressing demonstrated robust and comprehensive antibacterial properties, biofilm disruption efficacy, and augmented angiogenesis concomitant with PTT and NO gas therapy.

SEM observations shown in Fig. 2a confirm the successful transformation of PVA/CS/HTCC into uniform nanofibers, constituting a loose and porous nanofiber membrane. The addition of PANI and GSNO did not disrupt the porous structure of the nanofiber membrane. Under the encapsulation of these polymer fibers, GSNO is able to be released stably and uniformly. FT-IR spectroscopy corroborates the seamless integration of PANI and GSNO into the PCH nanofiber membrane, without initiating the formation of chemical bonds.

In Fig. 2d, e and Additional file 1: Fig. S6b, the PCH-based nanofiber membrane demonstrates a diminished water contact angle and an elevated water absorption ratio, indicating its aptitude for absorbing wound exudate. Previous research indicates that GA cross-linking enhances both the structural integrity and mechanical attributes of the PVA-CS-based nanofiber membrane [38]. This fortification ensures retention of structural coherence during water absorption, consequently amplifying the water absorption ratio [39]. This strategic design renders the nanofiber membrane dressings beneficial, facilitating augmented absorption of wound exudate and reducing the frequency of dressing changes.

The PCH-PANI-GSNO nanofiber membrane exhibited a pronounced photothermal response. When subjected to low-intensity NIR radiation at 0.5 W/cm^2^, the membrane’s temperature promptly rises to an approximate 58 °C, offering a significant edge in eradicating pathogenic bacteria (Fig. 2g). However, prolonged exposure to such thermal conditions may cause irreversible harm to adjacent skin tissues. Consequently, NIR irradiation is administered in a pulsatile manner. This regulated approach elevates the membrane’s temperature to a safer limit of approximately 56 °C, subsequently initiating a cooling phase. The temperature profile of the membrane remains stable across multiple on/off cycles (Fig. 2h), underscoring the reliable control and stability of PANI’s PTT efficacy.

The sustained bioactivity of drug-embedded wound dressings is desirable to reduce the frequency of dressing alterations. Historically, the majority of studies incorporating GSNO into biomaterials have observed a therapeutic release of NO spanning a duration from several hours to a week [40]. Electrospun nanofiber membranes, characterized by their high surface-to-volume ratio, increased porosity, and tunable physicochemical properties, have gained prominence in drug delivery research. Such membranes enhance the stability of therapeutic agents and maintain high localized drug concentrations at wound sites [41]. Several electrospun matrices have been shown to extend and modulate NO release [42, 43]. As depicted in Fig. 2i, the PCH-PANI-GSNO nanofiber membrane demonstrated a consistent release of NO for 10–12 days. Additionally, the electrospun membrane we have developed is adept at orchestrating bimodal release kinetics for nitric oxide gas: a prolonged, slow-release phase beneficial for angiogenesis and cellular migration is paired with a nanodetonator-like rapid release phase crucial for sterilization and biofilm disruption. This swift NO burst release is similar to the nanobombs-style NO release pattern previously reported by Yeonsu Jeong et al. [25]. The biocompatibility of wound dressings was validated through assays assessing cell viability, proliferation, and spreading activity. These assessments indicated that nanofiber membranes impregnated with HTCC, PANI, and GSNO at judiciously selected concentrations did not exert discernible effects (Additional file 1: Figs. S8-S10).

Impediments in localized angiogenesis and wound epithelialization are pivotal contributors to the prolonged non-healing trajectory observed in DW. Optimal wound dressings engineered for these specific pathologies should strategically facilitate the wound healing cascade, incorporating mechanisms to augment angiogenesis and expedite fibroblast chemotaxis [44]. Under the modulatory effect of NO [45, 46], all experimental cohorts equipped with a mechanism for controlled NO release—namely PCH-GSNO ± NIR, PCH-GSNO ± NIR—demonstrated marked enhancement in fibroblast chemotaxis and angiogenesis, as evidenced in Fig. 3 and Additional file 1: Fig. S12. Moreover, under the positive control of VEGF, the PCH-PANI-GSNO + NIR group demonstrated an angiogenic capability equivalent to 50 ng/mL of VEGF, showcasing the excellent performance of NO therapy in promoting angiogenesis.

Bacterial infection poses a significant impediment to the wound healing process. In this study, the PCH-PANI-GSNO nanofiber membrane was designed to swiftly and comprehensively eliminate primary pathogens implicated in diabetic wound infections, including MSSA, MRSA, and *E. coli*. The synergistic effects of photothermal therapy and a burst of NO facilitated the complete eradication of these bacterial strains within 5 min at initial concentrations of approximately 10^6^–10^8^ CFU/mL, including bacteria within biofilms (Figs. 4, 5 and Additional file 1: Figs. S13–14). The quaternary ammonium salt structure of HTCC, being positively charged, interacts with negatively charged phospholipid acids on bacterial cell walls, disrupting structural integrity and leading to cellular lysis. This endows the nanofiber membrane with a superior advantage in maintaining a non-infected local wound microenvironment compared to conventional dressings applied in clinical settings. Under physiological conditions, GSNO, uniformly distributed within the nanofiber membrane, releases NO in a sustained fashion. NO demonstrates bactericidal properties through lipid peroxidation and protein impairment mechanisms [47]. Additionally, NO interacts with superoxide radicals to form peroxynitrite and dinitrogen trioxide, compromising bacterial membrane integrity [47]. Under NIR irradiation, the PCH-PANI-GSNO nanofiber membrane, containing internal PANI rods, rapidly generates substantial heat, facilitating thermal bacterial eradication [48] and promoting swift GSNO decomposition within the membrane [25]. This subsequent NO burst, in concert with PTT, facilitates comprehensive elimination of both bacteria and biofilm in 5 min, building on the intrinsic antibacterial and anti-biofilm capabilities of the PCH-PANI-GSNO nanofiber membrane. In vivo antibacterial experiment data (Additional file 1: Fig. S16) reveal that, even compared to the PCH-PANI + NIR group, which also shows excellent antibacterial effects, the bacterial load in rats' wounds treated with PCH-PANI-GSNO + NIR is four orders of magnitude lower. This demonstrates that the synergistic action of PTT-induced NO burst release significantly enhances the antibacterial efficiency of PCH-PANI-GSNO nanofiber membranes. The synergy of PTT and NO therapy endows PCH-PANI-GSNO nanofiber membranes with robust antibacterial performance redundancy, making them highly capable of addressing complex application environments.

Figure 6 and Additional file 1: Fig. S17 delineates our endeavor to appraise the in vivo therapeutic potential of the PCH-PANI-GSNO nanofibrous membrane in augmenting the healing of DW. We constructed a diabetic wound model complicated by infection in male SD rats. Remarkably, a 14-day application of PCH-PANI-GSNO led to complete wound closure (Fig. 6c and Additional file 1: Fig. S17c). To evaluate the caliber of tissue repair, H&E and Masson’s trichrome staining were utilized (Fig. 7; Additional file 1: Fig. S17). The wounds subjected to PCH-PANI-GSNO nanofiber membranes’ treatment displayed the narrowest scar width. Moreover, treatment modalities enriched with NO, specifically PCH-GSNO ± NIR and PCH-PANI-GSNO ± NIR groups, resulted in wounds with enhanced epidermal thickness. Corroborating in vitro findings, the PCH-PANI-GSNO nanofiber membranes manifested an elevated vascular density relative to controls, underscoring the advantageous impact of sustained NO delivery on vascularization.

The Rap1 pathway, activated downstream of multiple surface receptors through guanine nucleotide exchange factors (GEFs), plays a pivotal role in regulating a wide range of fundamental cellular functions, including adhesion, migration, polarity, differentiation, and growth [49, 50]. Significant research on Rap1 in endothelial cells has delineated its critical role in the modulation of cadherin-based cell–cell junctions and in the regulation of vascular permeability [51]. Various GEFs have been identified to influence Rap1 activity in endothelial cells; for instance, increased cellular cAMP triggers Epac-mediated Rap1 activation, which is instrumental in promoting endothelial cell barrier integrity [49, 52, 53]. Moreover, the activation of C3G and PDZ-GEF, following interaction with cell–cell junction molecules such as cadherins and nectins, facilitates Rap1 activation in response to the dissociation of junctions [54]. Additionally, Rap1’s ability to activate the MAPK pathway across several cell types further highlights its versatile function [55]. The MAPK pathway, similar to the cAMP pathways, can regulate the promotion of keratinocyte proliferation and differentiation [56, 57], and it can also induce the Epithelial-to-mesenchymal transition process, thereby enhancing the migratory capabilities of epithelial cells [58] to promote wound healing. Similarly, the transcriptomic analysis of L929 cells suggests that PCH-PANI-GSNO nanofiber membranes may facilitate DW healing by modulating biological processes such as proliferation, differentiation, and cellular adhesion through the Rap1, MAPK, Cell adhesion molecules, and cAMP pathways. This highlights the potential therapeutic significance of PCH-PANI-GSNO nanofiber membranes, underscoring PCH-PANI-GSNO nanofiber membranes’ potential therapeutic importance.

The PCH-PANI-GSNO nanofibrous membrane has exhibited proficiency in mitigating infection, accelerating tissue repair, and augmenting cutaneous regeneration. Collectively, our in vivo and in vitro investigations substantiate that under the dual action of PPT and NO burst therapy, diabetic wounds can quickly move away from an infection-prone microenvironment that is adverse to healing. Moreover, the sustained and prolonged release of NO promotes the migration of fibroblasts and the formation of new blood vessels in the wound. These characteristics not only accelerate the process of wound healing and cutaneous reconstruction but also reduce the need for frequent dressing changes.

## Conclusion

In this study, aimed at enhancing the treatment of DW infections and overcoming perfusion obstacles, we developed a multifunctional PCH-PANI-GSNO nanofiber membrane wound dressing. The GSNO component within PCH-PANI-GSNO provides NO therapy, which promotes vascularization and wound healing in DWs, while the PANI component offers PTT therapy. This PTT therapy, combined with the thermally mediated burst release of NO from GSNO, facilitates the effective elimination of bacteria and biofilms through their synergistic action. This cost-effective, efficient multifunctional electrospun dressing introduces a new strategy for tackling the clinical challenges posed by diabetic wounds.

## Supplementary Information

**Additional file 1**. Supplementary information of additional methods, results, schematic illustrations, and figures.

## Data Availability

All data generated or analyzed during this are included in this paper.

## Abbreviations

DW: Diabetic wounds
PANI: Polyaniline
GSNO: S-Nitrosoglutathione
PVA: Poly(vinyl alcohol)
CS: Chitosan
HTCC: Hydroxypropyltrimethyl ammonium chloride chitosan
PTT: Photothermal therapy
PBS: Phosphate buffered saline
*E. coli*: *Escherichia coli*
MSSA: *Staphylococcus aureus*
MRSA: Methicillin-resistant *Staphylococcus aureus*
NO: Nitric oxide
NIR: Near-infrared
GA: Glutaraldehyde
HCl: Hydrochloric acid
DAPI: 4′,6-Diamidino-2-phenylindole
VEGF: Vascular endothelial growth factor
HUVECs: Human umbilical vein endothelial cells
SD: Sprague–Dawley
FT-IR: Fourier-transform infrared spectroscopy
SEM: Scanning electron microscopy
CFU: Colony-forming units
GO: Gene ontology
KEGG: Kyoto Encyclopedia of Genes and Genomes
H&E: Hematoxylin & Eosin
