# Supplementary material for: NIR-activated electrospun nanodetonator dressing enhances infected diabetic wound healing with combined photothermal and nitric oxide-based gas therapy
Source: J Nanobiotechnology. 2024 May 8;22:232. doi: 10.1186/s12951-024-02474-9 (PMC11546403; doi:10.1186/s12951-024-02474-9)
Supplement: Supplementary file 1 — Additional file 1. Supplementary information of additional methods, results, schematic illustrations, and figures. [file 12951_2024_2474_MOESM1_ESM.docx]

Supporting Information

*For*

**NIR-activated electrospun nanodetonator dressing enhances infected diabetic wound healing with combined photothermal and nitric oxide-based gas therapy**

*Jiajun Xie ^1^*^#^*, Guihua Liu ^2^*^#^*, Rong Chen ^1^*^#^*, Ding Wang* ^1^*, Huaming Mai ^1^, Qiang Zhong ^1^, Yanhong Ning ^1^, Jinlang Fu ^1^, Zinan Tang ^1^, Yixin Xu ^1^, Hao Li ^1^, Mingyuan Lei ^1^,* *Hao Cheng ^1^*，Yuliang Huang ^2^*，Yang Zhang ^1^**

^1^Division of Orthopaedic Surgery, Department of Orthopaedics, Nanfang Hospital, Southern Medical University, Guangzhou, Guangdong, 510515, PR China

^2^Institute of Orthopaedics, Huizhou Central People's Hospital, Huizhou, Guangdong, 516008, PR China

^#^These authors contributed equally to this work

Correspondence: *Hao Cheng ^1^*, *Yuliang Huang ^2^*, *Yang Zhang ^1^*

Tel +86-020-62787191

Fax 020-61641961

Dr. Hao Cheng, E-mail: haocool317@gmail.com

Prof. Yuliang Huang, E-mail: huangyl113@163.com

Prof. Yang Zhang, E-mail: [nfgjzy@126.com](http://nfgjzy@126.com)

***1. Methods***

***1.1 Exploratory experiments on the spinnability ratio of electrospun matrices***

The composite prepolymer formulations, designated as PVA : CS = 50% : 50%, PVA : CS = 60% : 40%, PVA : CS = 70% : 30%, PVA : CS = 80% : 20%, and PVA : CS : HTCC = 80% : 15% : 5%, each with a volume of 10 mL, were loaded into a 10 mL syringe fitted with a 22-gauge stainless-steel needle. The syringe was connected to the electrode of a high-voltage power supply unit (SIBEINING, China). Concurrently, the grounding wire was affixed to a drum collector rotating at a speed of 500 rpm. The electrospinning solution was extruded at a controlled flow rate of 5 μL/h via a precision syringe pump (SIBEINING, China). A gap of 16.5 cm was maintained between the needle tip and the collector, with the power supply unit set at a voltage of 22 kV. Subsequently, the resulting nanofibrous membranes were subjected to a vapor-phase crosslinking treatment using a mixture of glutaraldehyde (GA) and HCl for a duration of 1 hour at a pressure of 6 × 10-2 MPa within a vacuum chamber. The vapor-phase crosslinking agent was generated from 1 mL of 50 wt% GA and 20 µL of 37 wt% HCl in water, with HCl catalyzing the formation of acetal bridges between the hydroxyl groups of PVA and the aldehyde groups of GA. Post crosslinking, the membranes were allowed to air-dry in a fume hood for 24 hours to ensure the complete evaporation of residual GA and HCl. After ensuring the acquisition of morphologically uniform nanofiber membranes, the obtained nanofiber membranes were examined using a scanning electron microscope (Sigma 300, ZEISS, Germany).

***1.2 Selection of NIR irradiation power density***

The PCH-PANI-GSNO nanofibrous membrane was exposed to an 808 nm laser (LR-ISP-808, LASER TECHNOLOGY, China) irradiation at various power densities of 0.5, 1, 2 W/cm² for a duration of 120 seconds. The temporal evolution of temperature was monitored using an infrared thermal imaging camera (UTi 320E, UNI-T, China).

***1.3 SEM observation of nanofiber membranes after water absorption and degradation rate assessment***

To study the morphology of the nanofiber membranes after water absorption and degradation rate assessment, the scanning electron microscope (Sigma 300, ZEISS, Germany) was used to obtain microstructural images of the PCH-PANI-GSNO and PCH-PANI-GSNO + NIR groups' nanofiber membranes after water absorption and degradation rate assessment on days 3, 7, and 14.

***1.4 Selecting HTCC, PANI, and GSNO ratios in nanofibers via CCK-8 assay***

First, polymer premixes containing different ratios of HTCC (1.25, 2.5, 5, 10, 20, 30%), PANI (0.625, 1.25, 2.5, 5, 10, 20%), and GSNO (2.5, 5, 10, 15, 20, 25%) were prepared based on a PVA:CS composition of 80%:20%. Then, using the same method, nanofiber membrane discs (diameter 1cm, mass 10mg) were produced through electrospinning. Afterwards, under conditions of 37 °C and 5% CO₂, 1 × 10⁴ L929 cells were co-incubated with the aforementioned PC-based nanofiber membranes for periods of both 6 and 12 hours. Following the CCK-8 assay, the absorbance of the resulting supernatant was measured at a wavelength of 450 nm using a microplate reader (SpectraMax i3x, MOLECULAR DEVICES, Germany).

***1.5 Assessing rats’ wound bacterial burden via CFU count***

To clarify the bacterial burden of rat wounds and thereby validate the *in vivo* antibacterial performance of PCH-based nanofiber membranes, after treatment by each group, we collected tissue fluids from the rats' backs using swabs on days 3 and 7 after each group's treatment. The swabs were then washed with 1 mL of PBS to obtain bacterial solutions. After diluting these solutions by 5000 times, 20 μL of the diluted bacterial liquid from each group was plated. After incubating the agar plates at 37°C for 24 hours, the resulting CFUs were counted and assessed using ImageJ software (v1.53).

***2. Results***

***2.1*** ***Thickness of nanofiber membranes***

As shown in **Fig. S4g**, the thicknesses of the PCH, PCH-GSNO, PCH-PANI, and PCH-PANI-GSNO nanofiber membranes are respectively 451.00 ± 31.80 μm, 450.33 ± 20.21 μm, 458.33 ± 17.62 μm, and 447.33 ± 39.50 μm. There was no statistical difference in thickness among these groups.


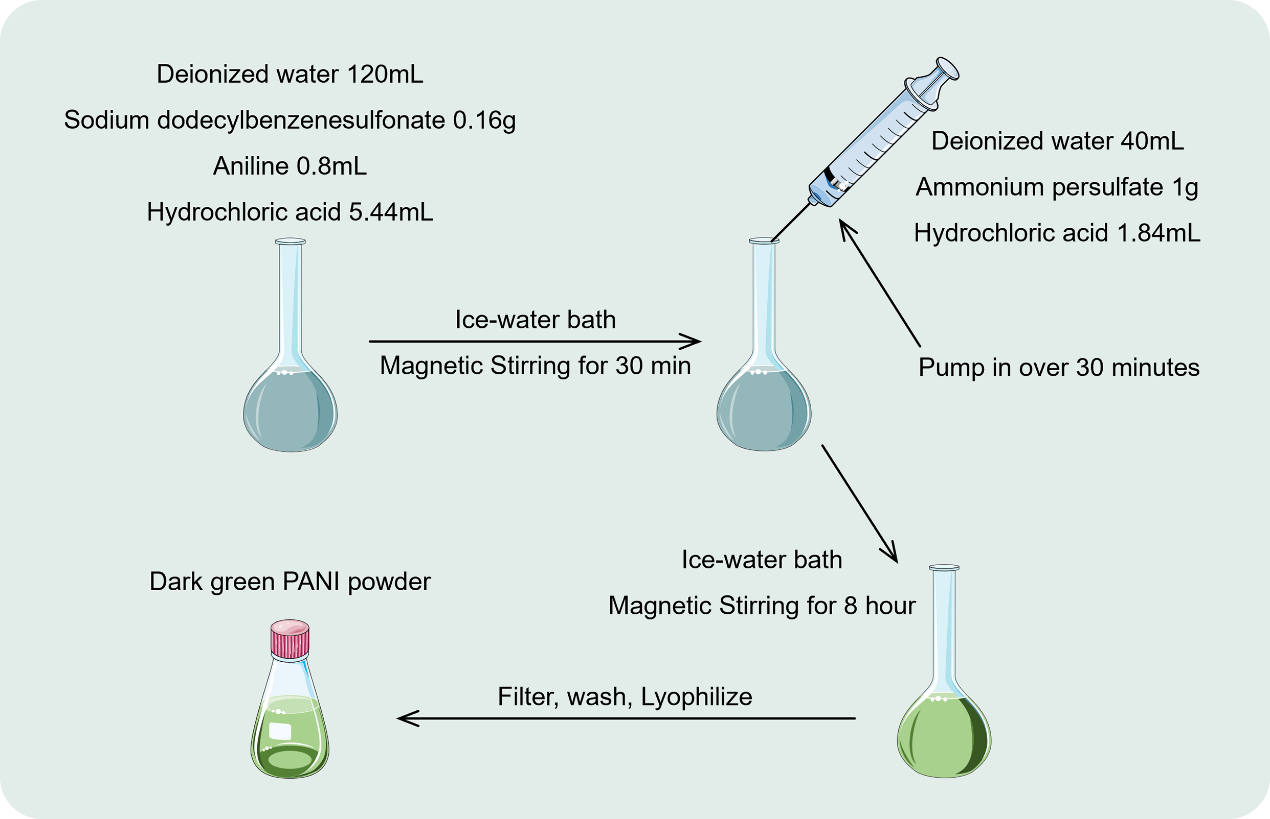


**Fig. S1.** Schematic illustration of the synthesis steps for PANI rods.


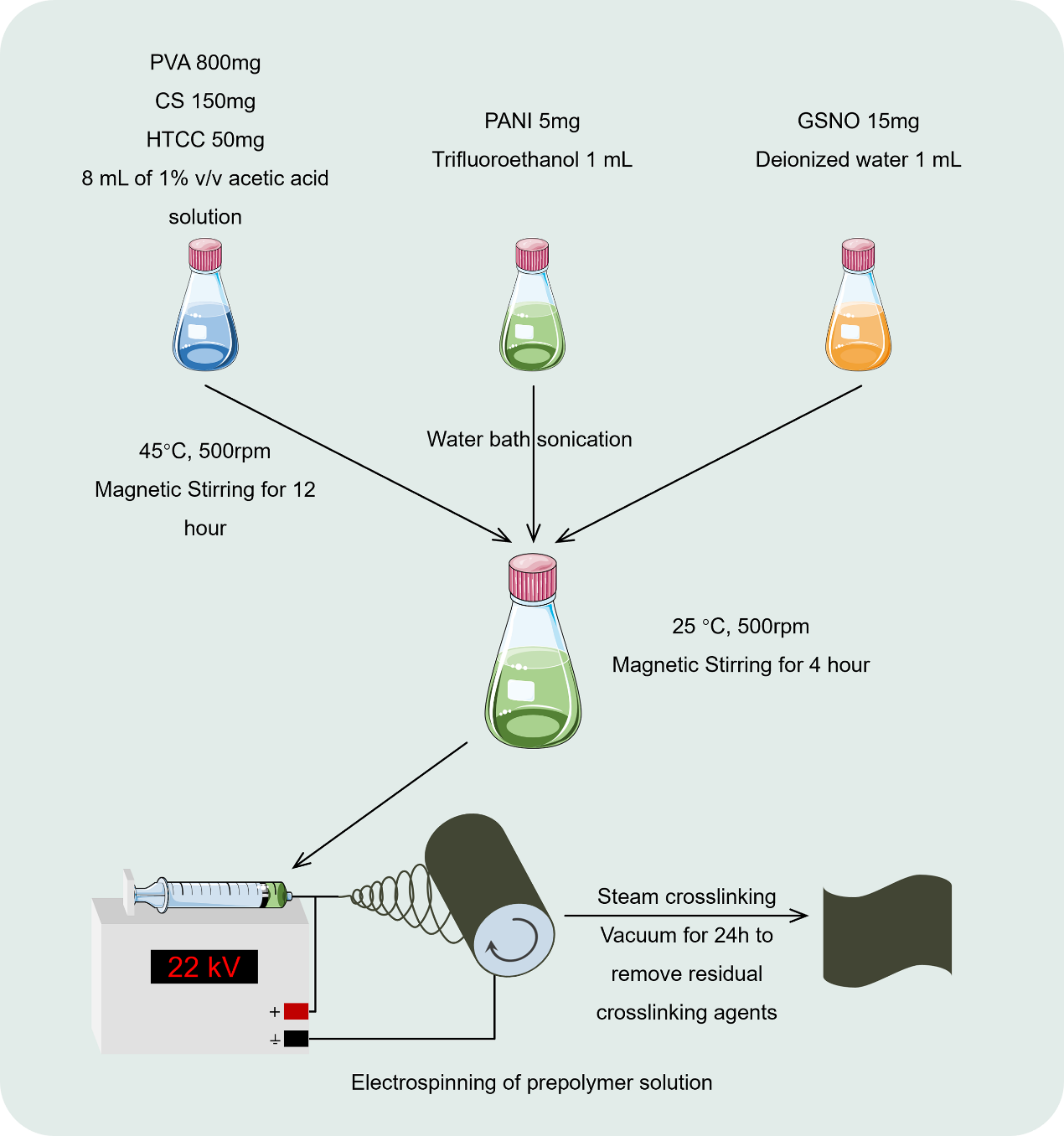


**Fig. S2.** Schematic illustration of the synthesis steps for PCH-PANI-GSNO nanofiber membrane.


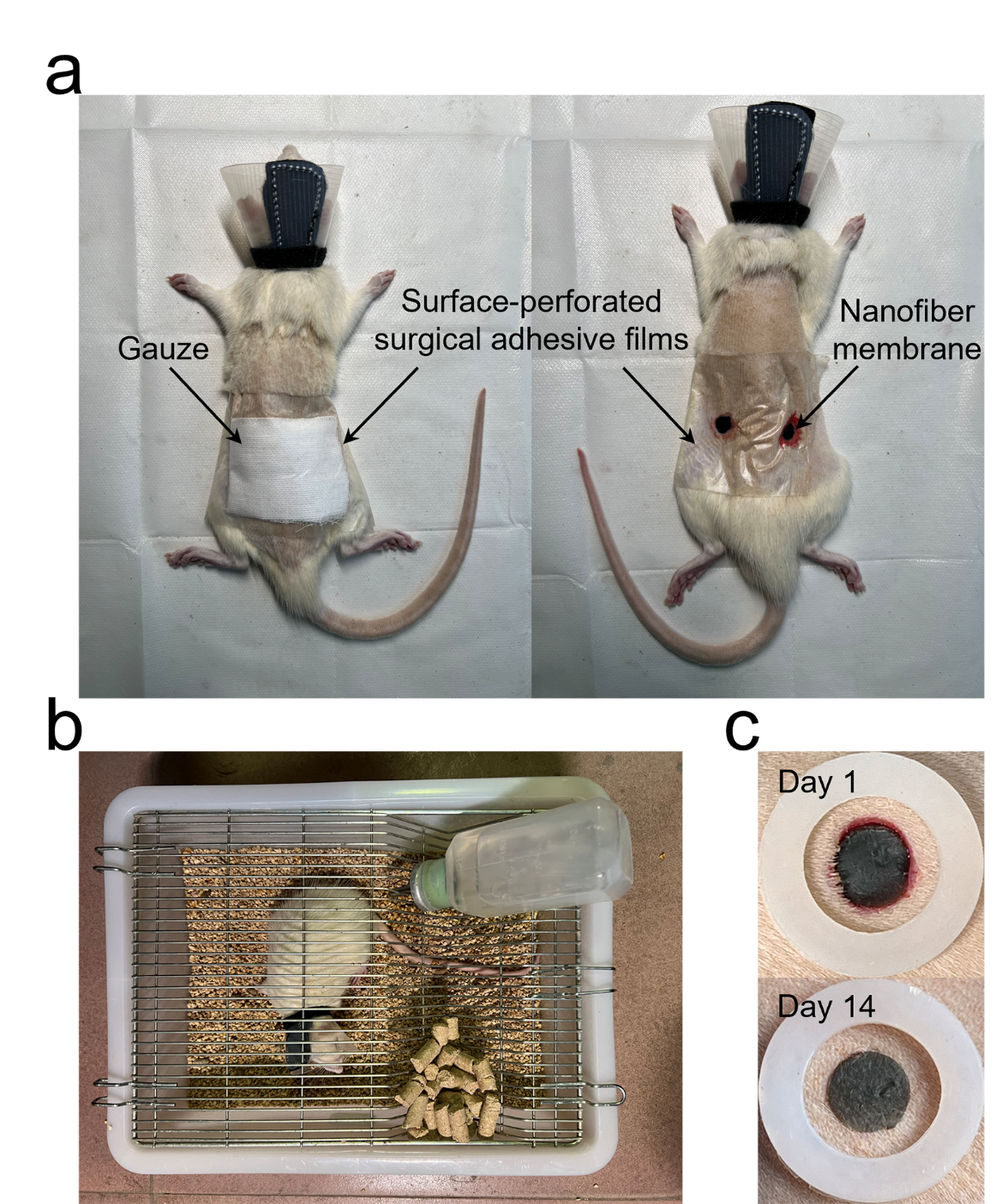


**Fig. S3.** Images illustrating the method of dressing fixation on rats and their housing conditions. (a) Different dressings were affixed to the dorsal wounds of rats using surface-perforated surgical adhesive films. (b) Rats were individually housed in cages with access to clean and readily available feed and water. (c) Photographs of rat wounds treated with PCH-PANI-GSNO nanofiber membranes on day 1 and day 14.


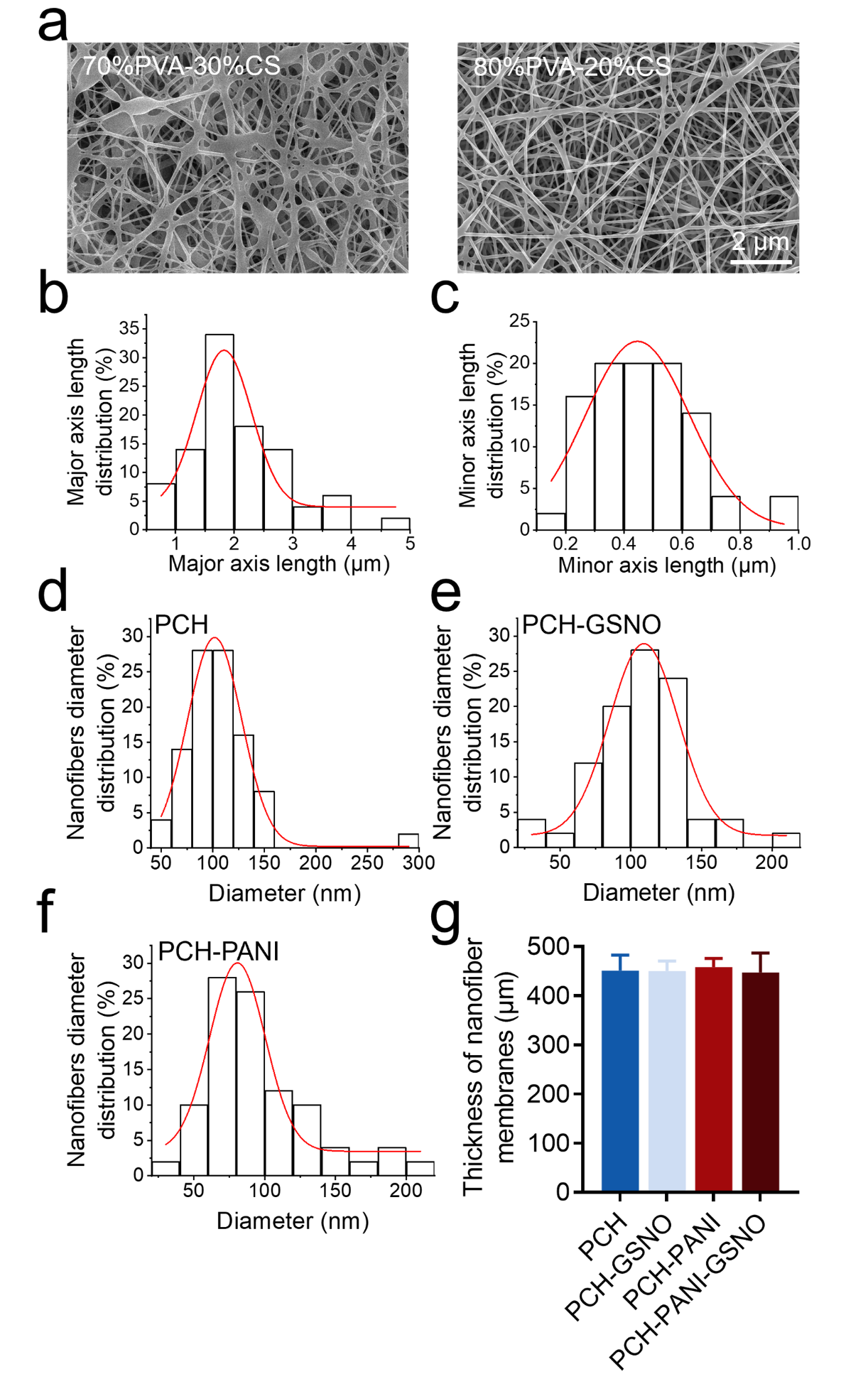


**Fig. S4.** Characterization of PANI rods and PCH-based nanofiber membranes. (a) SEM images of PVA-CS electrospun fibers at ratios of 70% PVA to 30% CS and 80% PVA to 20% CS. (b-c) Major axis length and Minor axis length distribution of PANI rods. (d-f) The diameter distribution of PCH, PCH-GSNO and PCH-PANI nanofibers. (g) Thickness measurement data for PCH, PCH-GSNO, PCH-PANI, and PCH-PANI-GSNO nanofiber membranes. (n = 3)


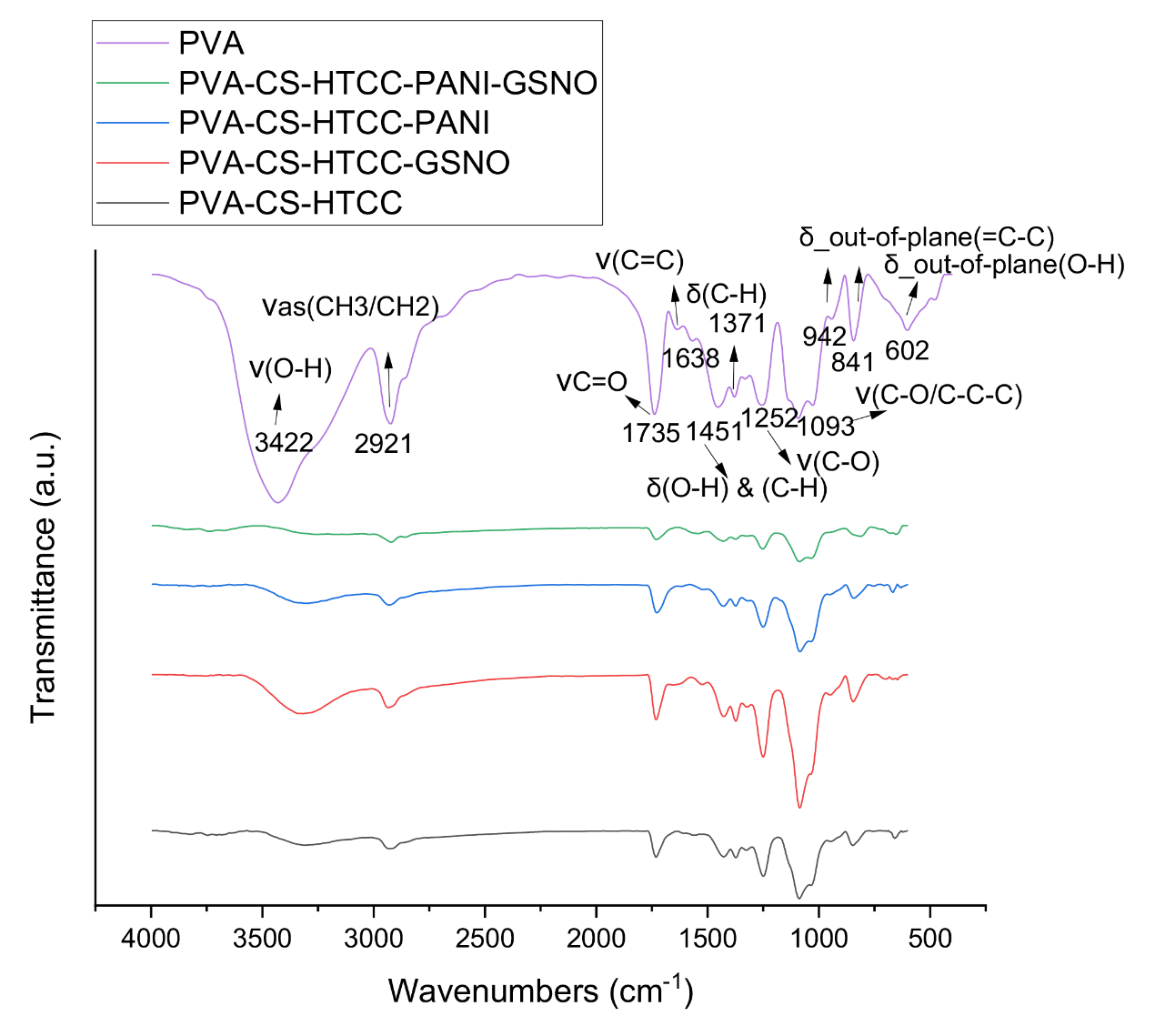


**Fig. S5.** FT-IR spectra for PVA, PCH, PCH-GSNO, PCH-PANI, and PCH-PANI-GSNO.


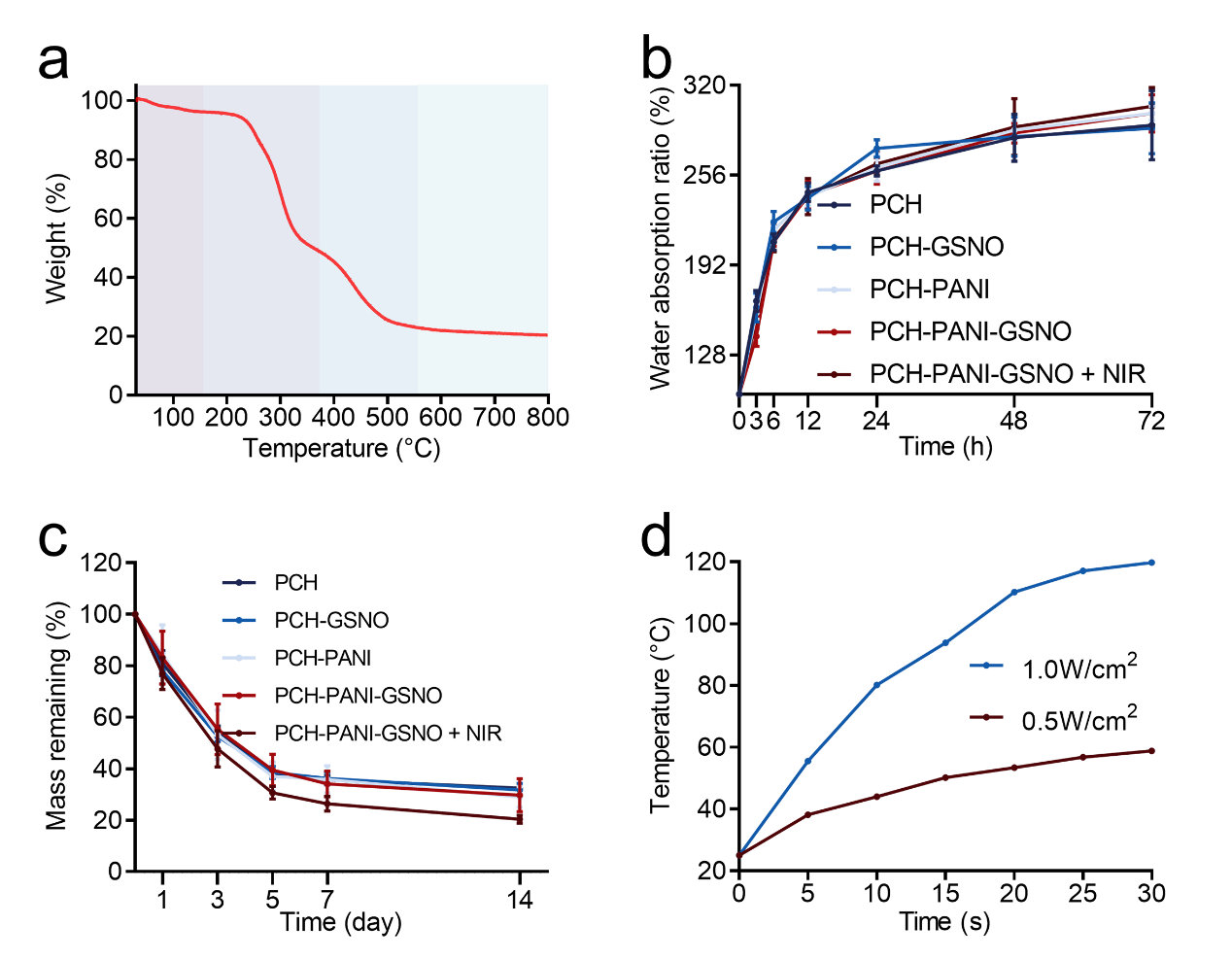


**Fig. S6.** Characterization of PCH-based nanofiber membranes. (a) Thermogravimetric analysis of PCH-PANI-GSNO nanofiber membrane. (b) Water absorption curves of PCH-based nanofiber membranes. (c) Water absorption curves of PCH-based nanofiber membranes within 3 days. (d) *In vitro* degradation curves of PCH, PCH-GSNO, PCH-PANI, and PCH-PANI-GSNO nanofiber membranes within 14 days.(n = 3)


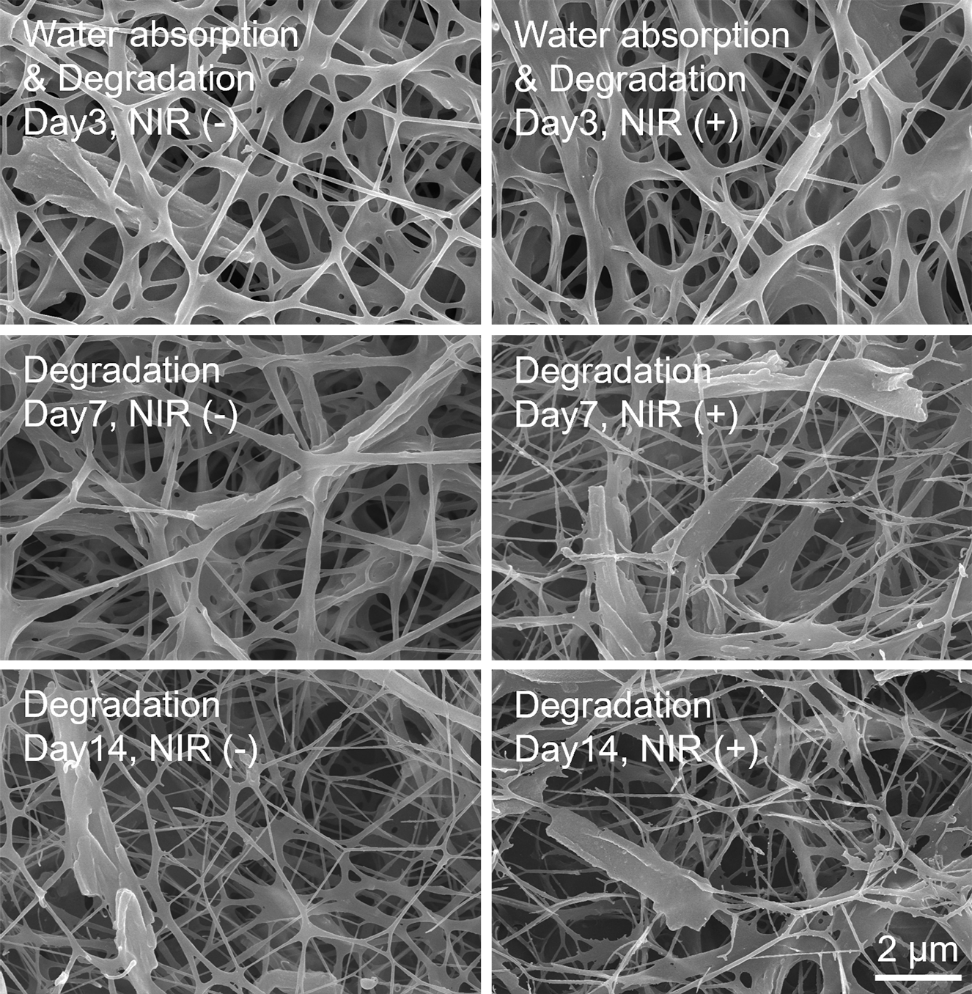


**Fig. S7.** SEM images of the morphological changes in PCH-PANI-GSNO nanofiber membranes during the water absorption and degradation experiments.


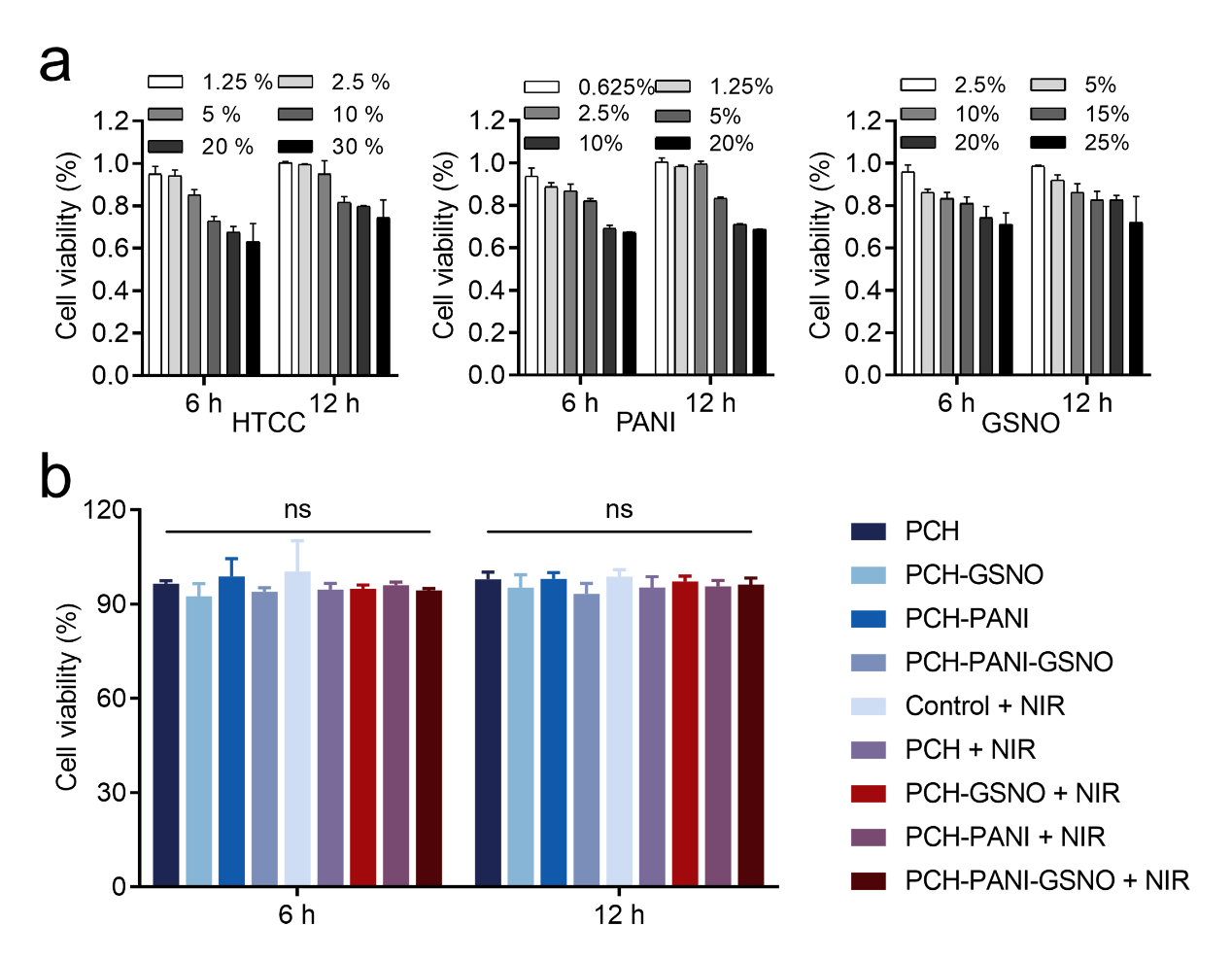


**Fig. S8.** The composition load ratios within PCH-based nanofiber membranes and the biocompatibility of the PCH-based nanofiber membranes were determined through CCK-8 assays. (a) Nanofiber membranes made of PVA-CS and infused with diverse concentrations of HTCC, PANI, and GSNO underwent co-culture with L929 cells, followed by CCK-8 assays. (b) L929 cells, post-treatment with different PCH-based nanofiber membranes, were subjected to CCK-8 assays.


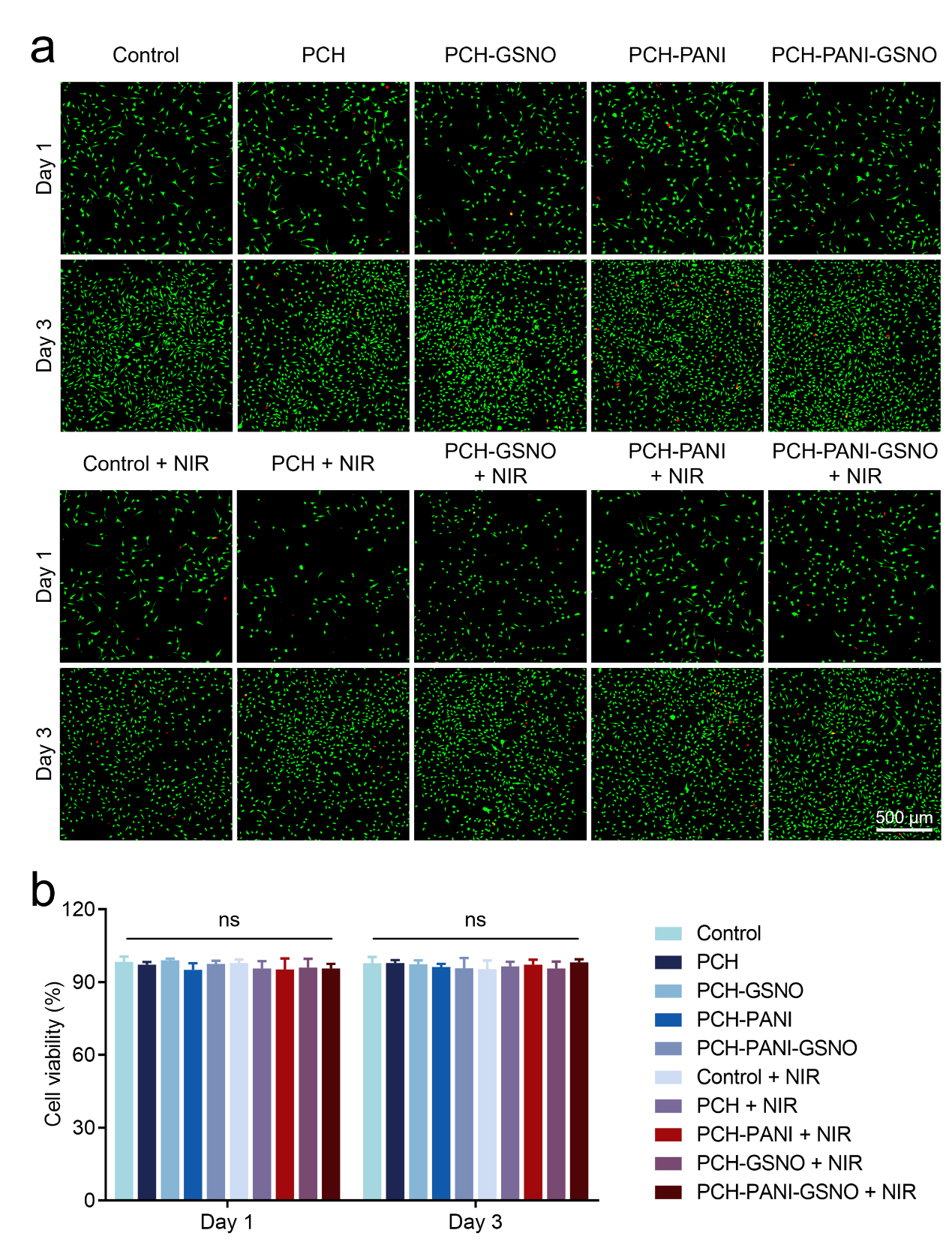


**Fig. S9.** The biocompatibility of PCH-based nanofiber membranes was evaluated using live/dead cell staining. (a) Images of L929 cells, observed after live/dead cell staining, on days 1 and 3 following co-incubation with PCH-based nanofiber membranes, signifying live cells in green and dead cells in red. (b) Cell viability of L929 cells post-co-incubation with PCH-based nanofiber membranes was assessed by calculating the ratio of live cells. (Three biological replicates; images from three fields of view each)


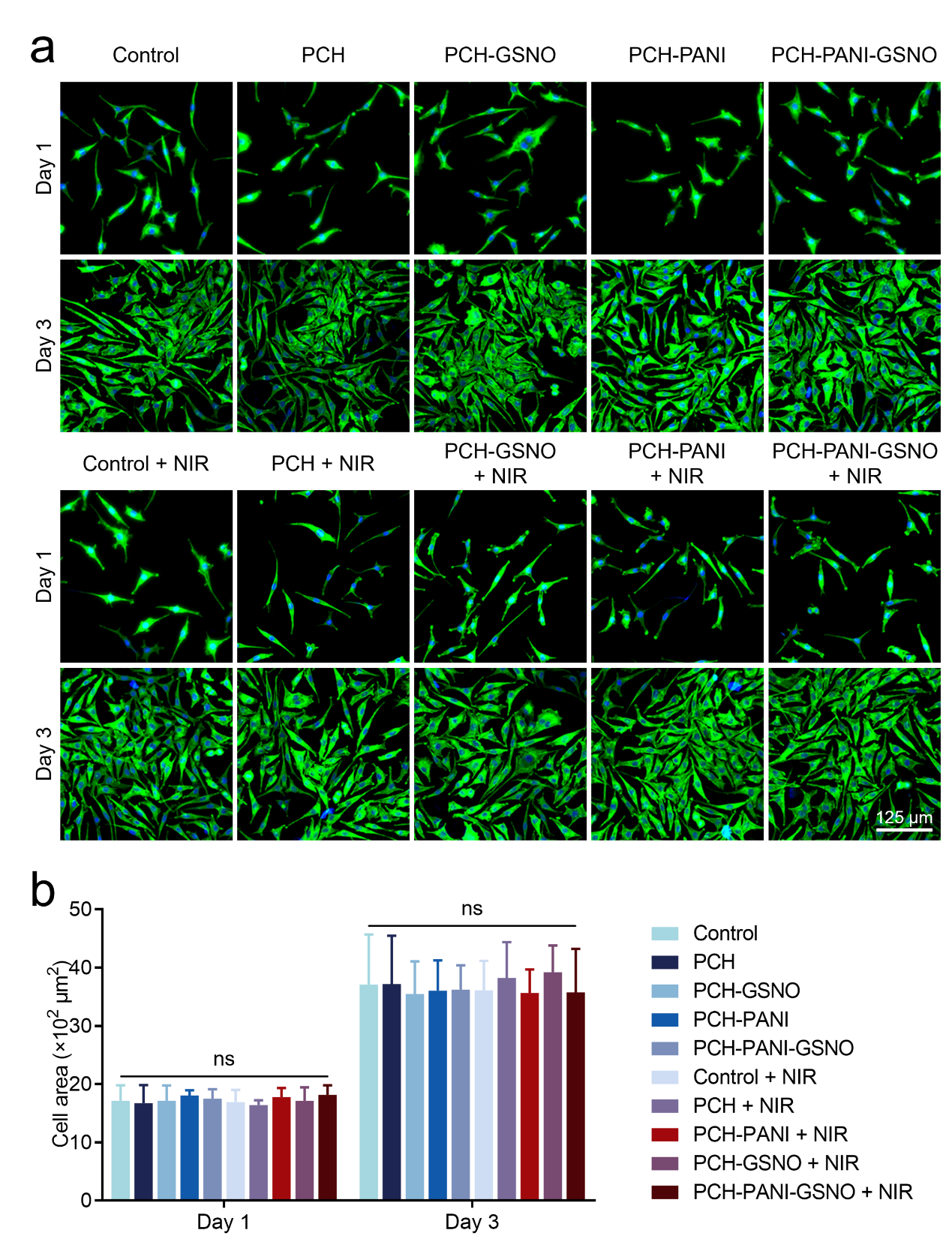


**Fig. S10.** The biocompatibility of PCH-based nanofiber membranes was evaluated using cell spreading area assessment. (a) L929 cells were visualized using phalloidin (green for actin filaments) and DAPI (blue for nuclei) stains on days 1 and 3. (b) Quantitative analysis of the average cell area of L929 cells on days 1 and 3 after co-incubation with PCH-based nanofiber membranes. (Three biological replicates; images from three fields of view each)


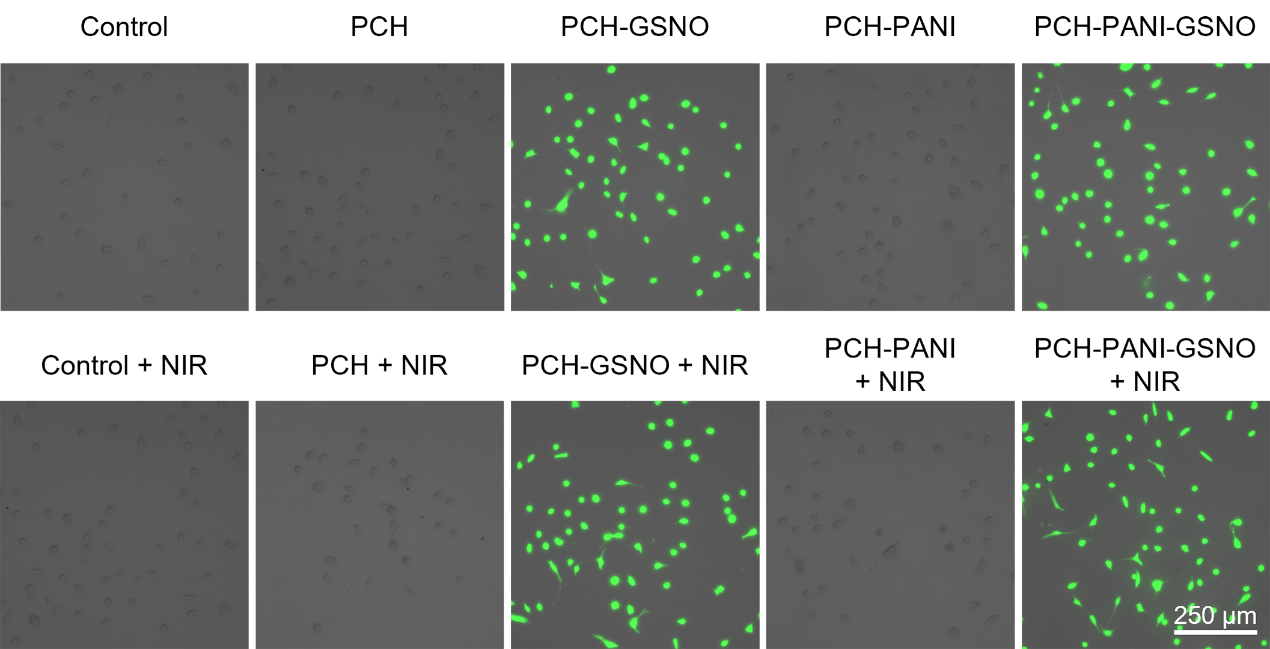


**Fig. S11.** Fluorescence microscopic images of L929 cells post co-culture with various PCH nanofiber groups and subsequent application of 4-Amino-5-Methylamino-2',7'-Difluorofluorescein Diacetate.


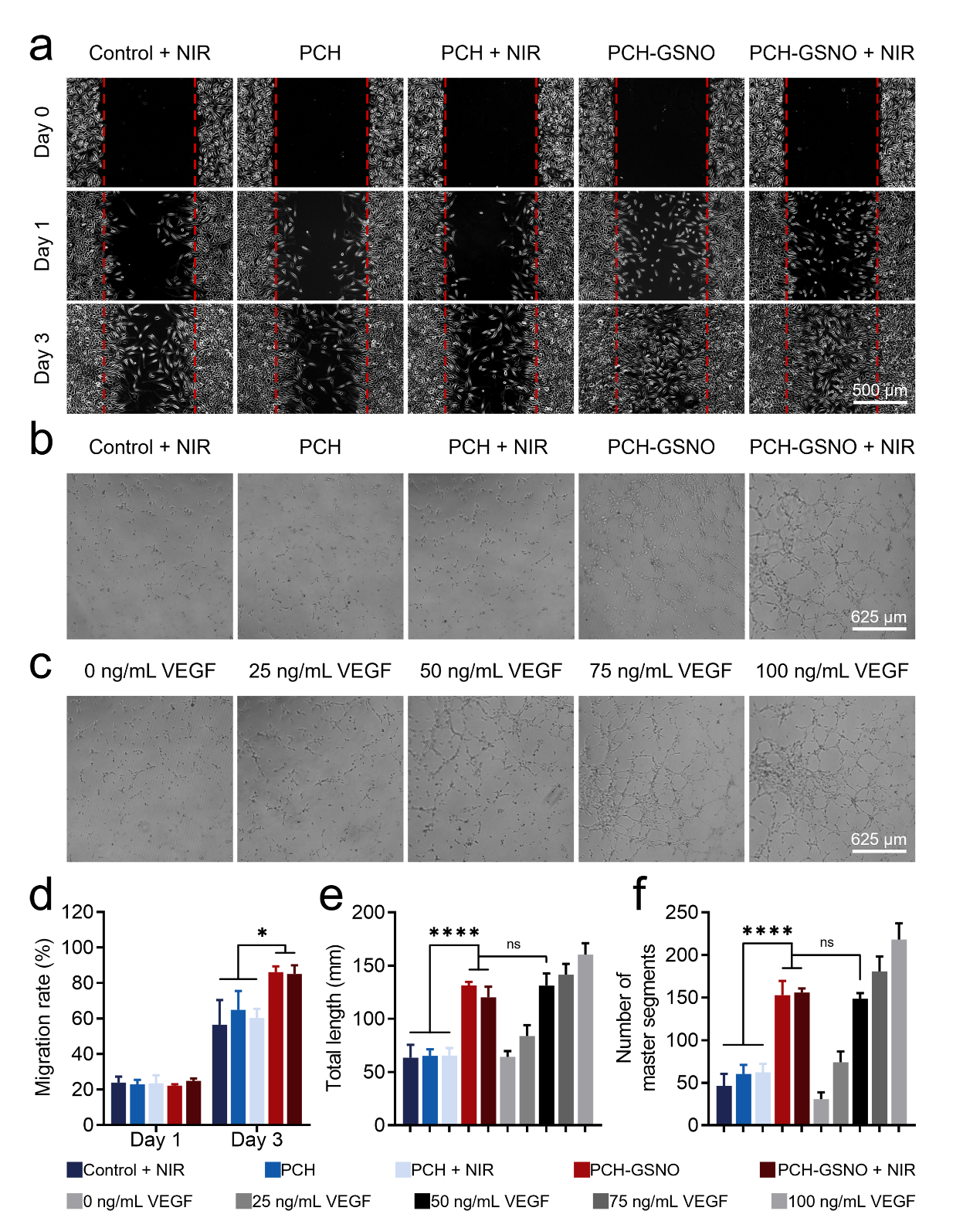


**Fig. S12.** Analysis of the promotion of cell migration and angiogenic properties of the PCH-based nanofiber membrane. (a, d) The scratch assay outcomes and analysis of the migration rate for L929 cells post-treatment with PCH-based nanofiber membranes on days 0, 1, and 3. (b-c, e-f) The tube-forming ability of HUVECs after treatment with PCH-based nanofiber membranes and various concentrations of VEGF. (Three biological replicates; images from three fields of view each; ^∗^*P* < 0.05, ^∗∗^*P* < 0.01, ^∗∗∗^*P* < 0.001, ^∗∗∗∗^*P* < 0.0001).


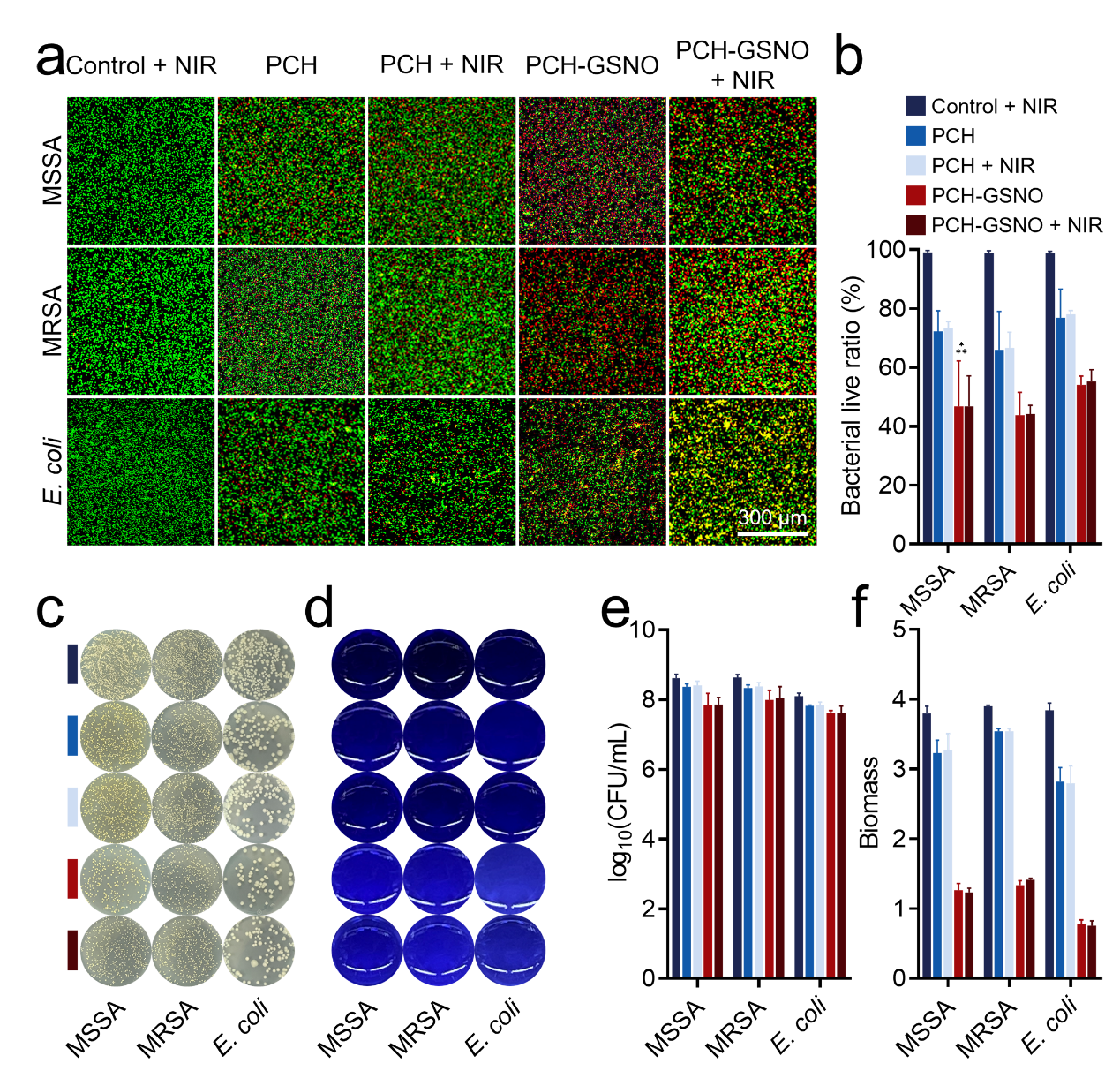


**Fig. S13.** Evaluation of the antibacterial properties of PCH-based nanofibrous membranes *in vitro*. (a-b) Bacterial live/dead staining of MSSA, MRSA and *E. coli* post-treatment with PCH-based nanofiber membranes and subsequent live bacteria ratio quantification. (c, e) The CFU counting method tests the *in vitro* antibacterial effects of PCH-based nanofiber membranes and the quantitative analysis of CFU. (d, f) Biofilm formation by MSSA, MRSA, and *E. coli* visualized using crystal violet staining post diverse treatments, and the OD590 value of the biofilms from the three bacteria following crystal violet staining. (Three biological replicates; images from three fields of view each; ^∗^*P* < 0.05, ^∗∗^*P* < 0.01, ^∗∗∗^*P* < 0.001, ^∗∗∗∗^*P* < 0.0001).


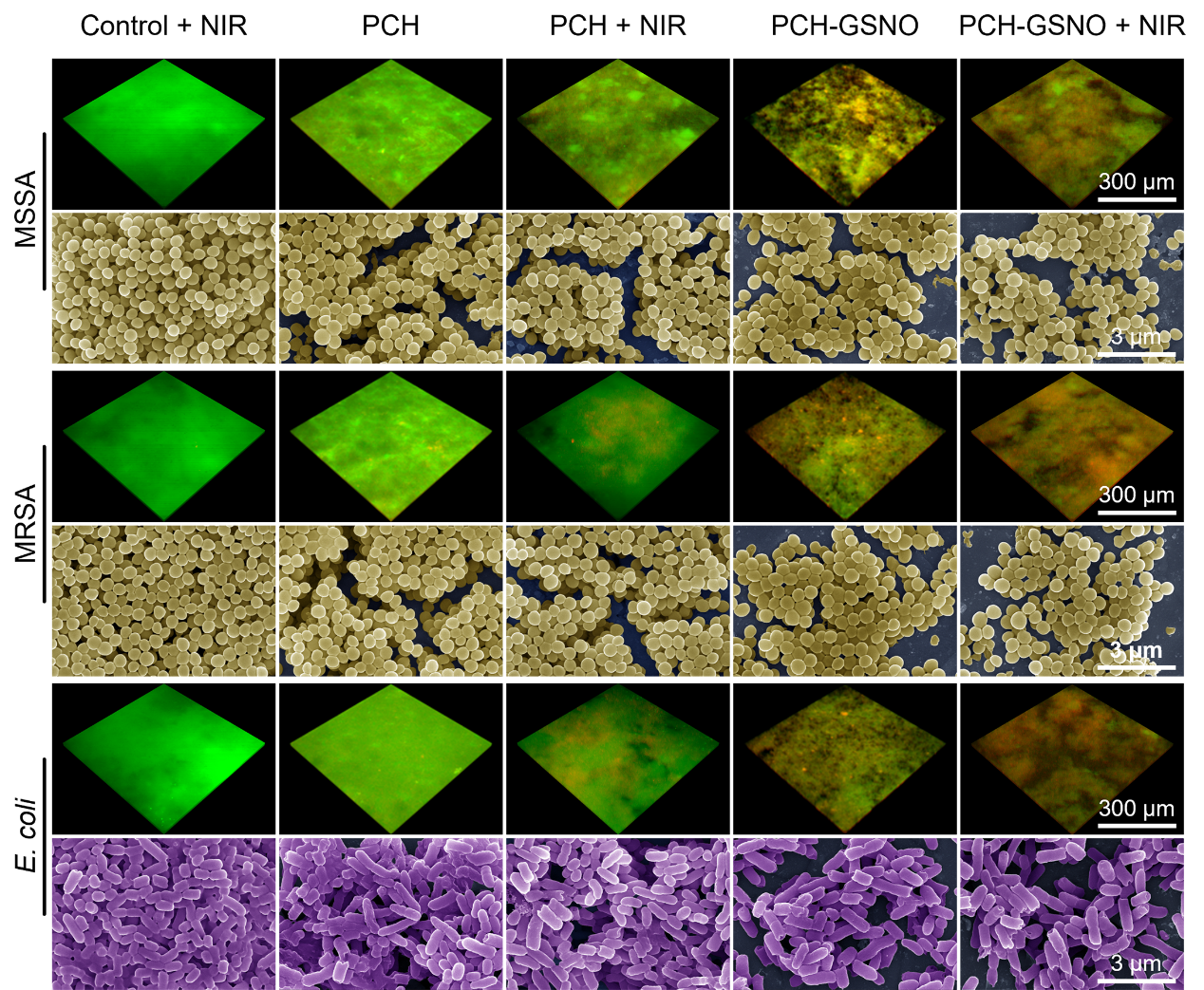


**Fig. S14.** Biofilms response to control, PCH-PANI, PCH-PANI+NIR, PCH-PANI-GSNO, PCH-PANI-GSNO+NIR treatment in SEM and Z-stack scanning. Z-stack scanning from live/dead staining and SEM images of biofilms produced by three types of bacteria (MSSA, MRSA, and *E. coli*) after treatment with PCH-based nanofiber membranes.


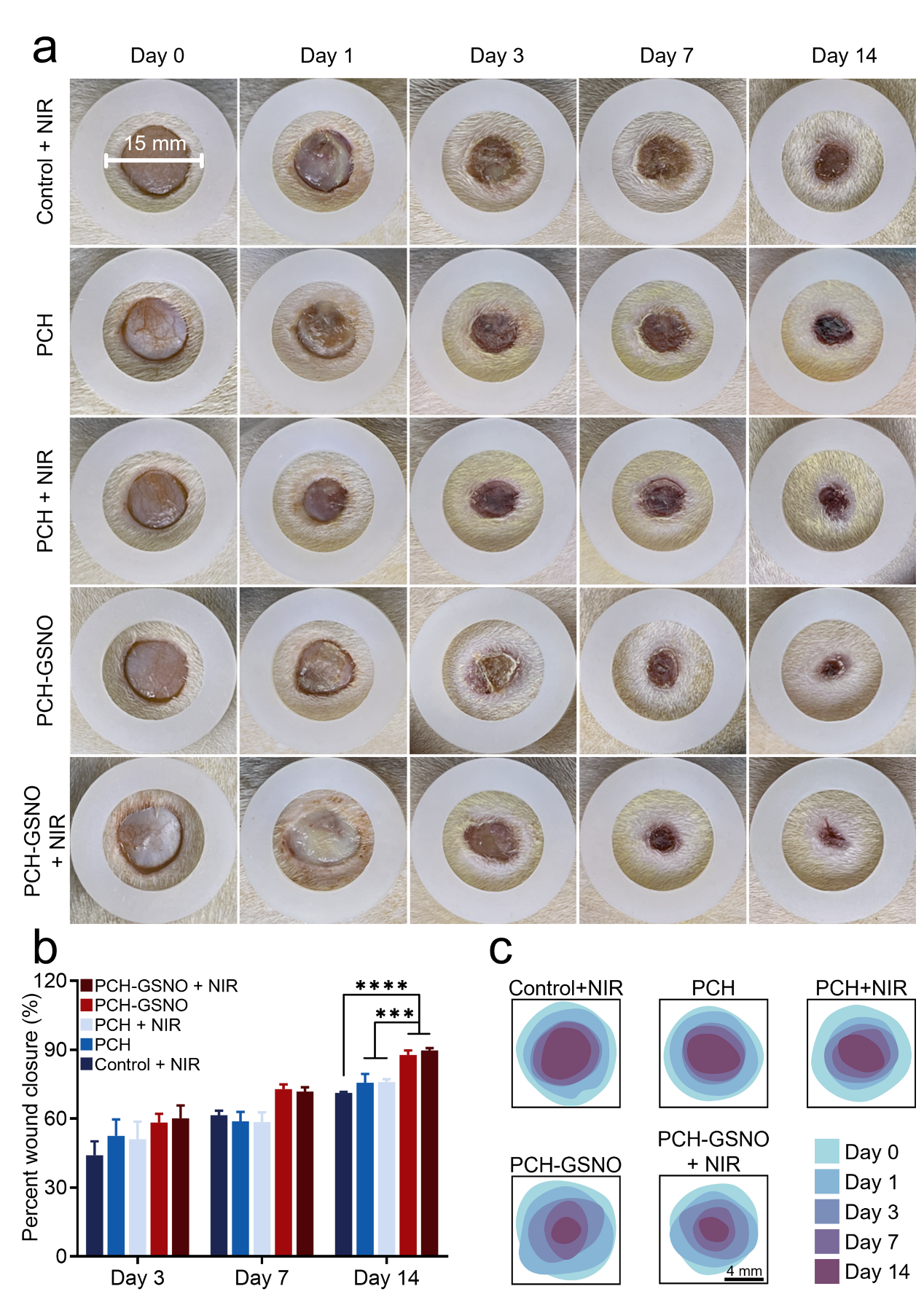


**Fig. S15.** PCH-based nanofiber membranes promoted infected diabetic wound healing. (a) Images depicting wound contraction dynamics on the wound site for the control, PCH-PANI, PCH-PANI + NIR, PCH-PANI-GSNO, and PCH-PANI-GSNO + NIR groups on days 0, 1, 3, 7, and 14. (b) Quantitative analysis of the wound healing rate on days 3, 7, and 14. (c) Simulation of wound dynamics over the 14-day treatment in the control, PCH-PANI, PCH-PANI + NIR, PCH-PANI-GSNO, and PCH-PANI-GSNO + NIR groups groups. (n = 3; ^∗^*P* < 0.05, ^∗∗^*P* < 0.01, ^∗∗∗^*P* < 0.001, ^∗∗∗∗^*P* < 0.0001).


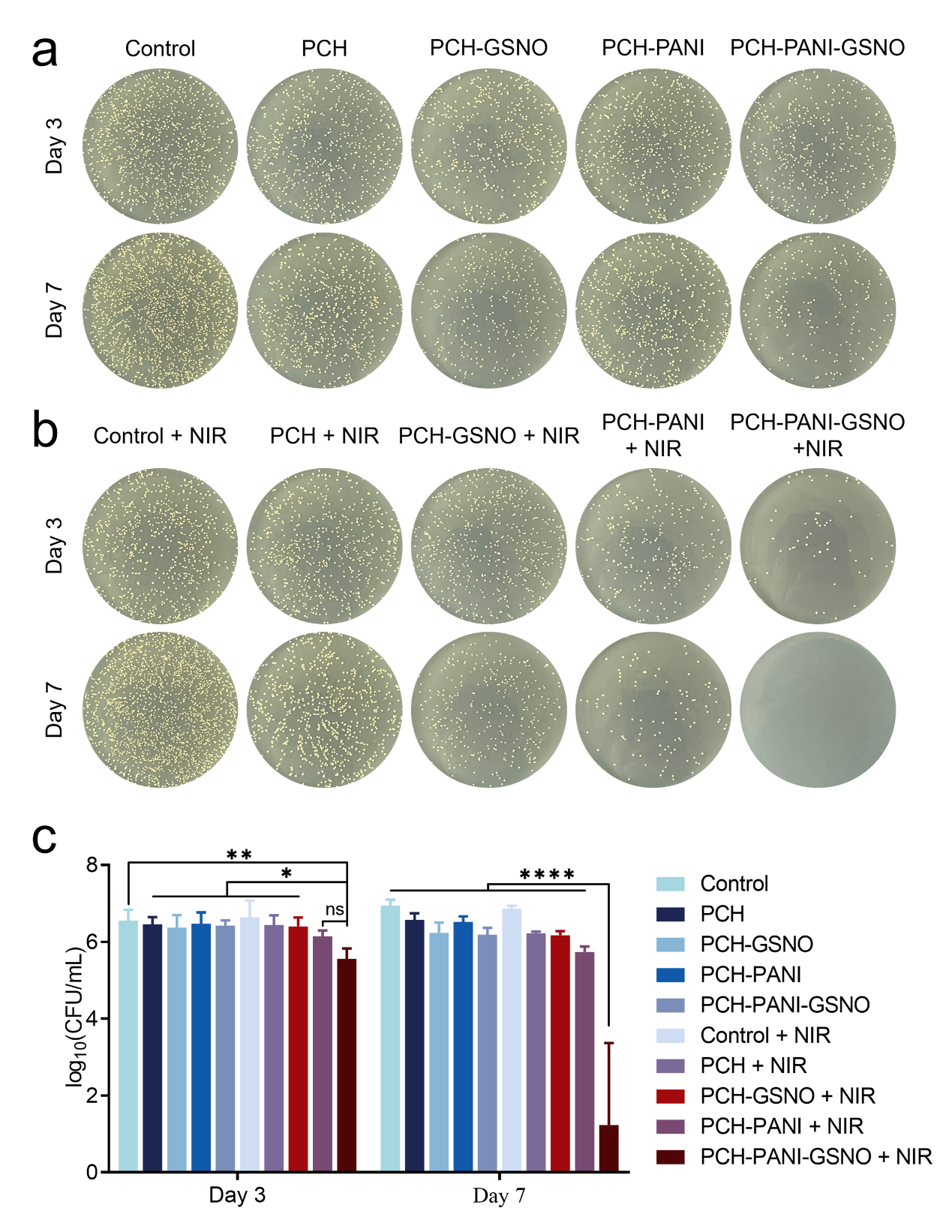


**Fig. S16.** *In vivo* antibacterial abilities of PCH-based nanofiber membranes against methicillin-resistant *Staphylococcus aureus* (MRSA). (a-b) Images of bacterial colony counts cultured on agar plates, obtained from dilutions of wound tissue fluid following various treatments on days 3 and 7. (c) The quantitative analysis of the CFU in wound tissue fluid after treatment in various groups. (^∗^*P* < 0.05, ^∗∗^*P* < 0.01, ^∗∗∗^*P* < 0.001, ^∗∗∗∗^*P* < 0.0001).


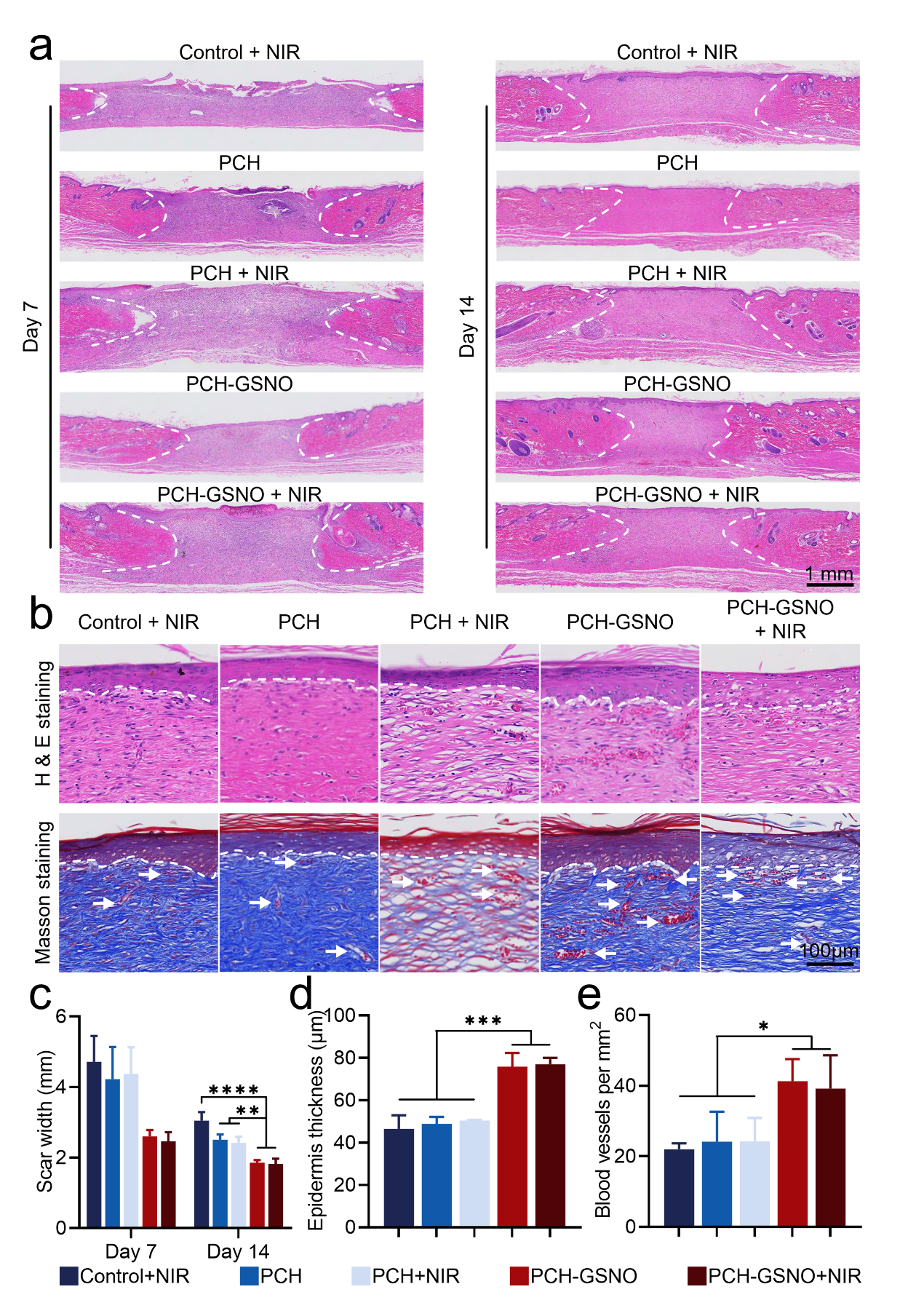


**Fig. S17.** Assessment of wound repair via H&E and Masson staining. (a, c) Results of healing wounds on days 7 and 14, represented by H&E staining. White dashed lines mark unhealed scars. Scar widths for various groups analyzed with ImageJ (v1.53) (b) Day 14 skin samples' H&E and Masson-stained images. Dashed lines denote the epidermis, and arrows point to blood vessels. (d) Epidermal thickness analysis of wounds after nanofiber membrane treatment using ImageJ on day 14 skin samples. (e) Quantification of blood vessels at wound locations via ImageJ on day 14 skin samples. (n = 3; ^∗^*P* < 0.05, ^∗∗^*P* < 0.01, ^∗∗∗^*P* < 0.001, ^∗∗∗∗^*P* < 0.0001).


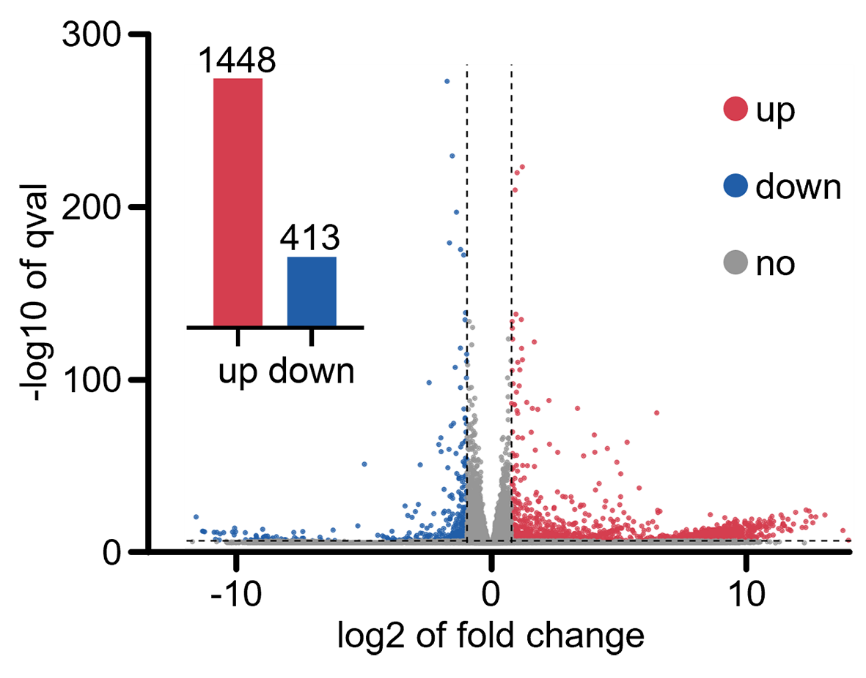


**Fig. S18.** Distribution of DEGs in L929 cells under treatment with PCH-PANI-GSNO + NIR and control group.
